# Diagnostic and Prognostic Value of Hematological Parameters in Necrotizing Enterocolitis: A Systematic Review

**DOI:** 10.3390/jcm14072530

**Published:** 2025-04-07

**Authors:** Rozeta Sokou, Petros Mantzios, Alexia Eleftheria Palioura, Andreas G. Tsantes, Alexandra Lianou, Daniele Piovani, Konstantina A. Tsante, Katerina Lampropoulou, Nicoletta Iacovidou, Stefanos Bonovas

**Affiliations:** 1 Neonatal Department, National and Kapodistrian University of Athens, Aretaieio Hospital, 11528 Athens, Greece; niciac58@gmail.com; 2 Immunology and Histocompatibility Department, Evangelismos General Hospital, 10676 Athens, Greece; petrosmantzios@gmail.com; 3 Neonatal Intensive Care Unit, “Agios Panteleimon” General Hospital of Nikea, 18454 Piraeus, Greece; al.palioura@gmail.com (A.E.P.); alexlianou95@gmail.com (A.L.); 4 Microbiology Department, “Saint Savvas” Oncology Hospital, 11522 Athens, Greece; andreas.tsantes@yahoo.com; 5 Department of Biomedical Sciences, Humanitas University, Pieve Emanuele, 20072 Milan, Italy; dpiovani@hotmail.com; 6 IRCCS Humanitas Research Hospital, Rozzano, 20089 Milan, Italy; 7 Department of Biomedical Sciences, University of West Attica, 12243 Athens, Greece; ktsante@yahoo.com; 8 Neonatal Intensive Care Unit, School of Medicine, University of Ioannina, 45110 Ioannina, Greece; katerina.lambropoulou@yahoo.gr

**Keywords:** necrotizing enterocolitis, complete blood count, neonates, anemia, white blood cells, platelets

## Abstract

**Background/Objectives**: Necrotizing enterocolitis (NEC) is a severe, potentially fatal gastrointestinal disease that primarily affects preterm neonates, especially those with very low birth weight (<1500 g). Despite extensive research, its pathophysiology remains unclear, with NEC considered a spectrum of disorders driven by systemic inflammation, microbiota dysregulation, and intestinal hypoxic injury. Diagnosis is challenging due to its subtle presentation and reliance on clinical and radiographic findings, underscoring the urgent need for reliable early biomarkers. Complete blood count (CBC) is one of the most frequently performed laboratory tests in neonatal care, providing valuable insights associated with hematologic alterations associated with NEC. Given its cost-effectiveness, accessibility, and rapid turnaround time, CBC parameters have been increasingly investigated for their diagnostic and prognostic potential in NEC. This systematic review consolidates existing evidence on the diagnostic and prognostic utility of CBC parameters in NEC, examining their association with disease onset, progression, and outcomes. **Methods**: A systematic review of the literature in PubMed and Scopus databases was conducted, between February 25 and December 2024. **Results**: Following a PRISMA-compliant search strategy, 77 eligible studies were included, analyzing data from 295,195 neonates, of whom 14,570 had NEC. Among the 77 studies, 17 examined NEC-associated mortality as a primary outcome, while 13 studies focused on the development of predictive models incorporating CBC parameters alongside other clinical and laboratory data to assess NEC severity and prognosis in neonates. The findings highlight the potential of CBC-derived markers to facilitate early NEC detection and risk stratification. However, variations in study design and diagnostic criteria highlight the need for prospective studies to validate their clinical use. **Conclusions**: Despite advancements in understanding NEC, its diagnosis remains challenging due to the absence of fully reliable biomarkers. CBC parameters show promise in offering early diagnostic and prognostic insights. However, further validation is needed for their routine integration into NICU practice. Given the persistent challenges in NEC diagnosis and management, our findings highlight the necessity for integrated scoring systems that combine hematologic, clinical, and radiologic data to enhance early detection and optimize neonatal care. Further research is essential to refine these predictive models, enabling timely interventions and improving survival rates in NEC-affected neonates.

## 1. Introduction

Necrotizing enterocolitis (NEC) is a severe gastrointestinal disease that primarily affects preterm neonates (85–93% of cases), particularly those with very low birth weight (VLBW, <1500 g), with a prevalence exceeding 7% in this group. NEC carries a high mortality rate of 15–30%, especially when surgical intervention is necessary [1,2]. Severe NEC (defined as Stage ≥ 2 according to modified Bell’s criteria) is associated with multiple long-term complications, including GI strictures, short bowel syndrome, nutrient malabsorption and neurocognitive impairment [1,3,4]. In term neonates, NEC is much rarer and is typically associated with pre-existing conditions such as congenital heart disease, congenital gastrointestinal malformations (e.g., gastroschisis, Hirschsprung’s disease), neonatal hypoxemia, neonatal hyperglycemia, and certain maternal conditions. In this population, the reported incidence varies between 1 case per 20,000 births and 10–13% of all NEC cases [5,6]. Studies have highlighted the frequency of underlying etiologies in term infants with NEC. Both maternal and perinatal characteristics are documented risk factors for NEC development. In preterm neonates, factors such as gut immaturity, enteral feeding, microbial dysbiosis, local ischemia and/or reperfusion injury, and inflammation are believed to play key roles in the development of NEC [7,8]. On the other hand, breast feeding serves as a known protective factor [9]. The critical role of intestinal immaturity is highlighted by the strong inverse correlation between gestational age (GA) and both the incidence of NEC and its associated mortality. NEC typically occurs between the 2nd and 3rd week of life, although in some infants, onset can be delayed up to 3 months. Early-onset NEC (EO-NEC) is primarily linked to inflammatory and perinatal factors, while late-onset NEC (LO-NEC) mostly associated to intestinal microbiota dysbiosis and infant feeding practices. The timing of onset correlates with disease severity, with early-onset NEC generally exhibiting a more severe course and worse clinical outcomes compared to late-onset NEC [10]. In full-term infants, LO-NEC is defined as the development of the disease after the 7th day of life, while for preterm infants, there is no clear definition/distinction for early and late onset NEC. Differences in the clinical presentation and course of NEC are evident, with EO-NEC having a more severe and rapid progression, while LO-NEC typically presents with milder symptoms and slower progression, often requiring less invasive treatment [11]. Evidence support the hypothesis that EO-NEC and LO-NEC exhibit significant differences in both clinical, laboratory and microbiological characteristics [10]. These differences extend to hematological profiles, with early-onset NEC often presenting with neutropenia and thrombocytopenia as a result of perinatal stress, whereas late-onset NEC is typically characterized by progressive thrombocytopenia and leukocytosis, indicating an ongoing inflammatory process [12,13].

Despite extensive research over the years, NEC remains a largely enigmatic disease. Its exact nature and pathophysiological mechanisms are not fully understood. However, the prevailing view is that NEC encompasses a complex group of conditions that can emerge from various pathological processes. There is growing recognition that the term “NEC” may refer to a spectrum of distinct disorders with varying underlying mechanisms, some of which result in intestinal necrosis. Furthermore, research has identified different predisposing factors in preterm and term neonates [14,15]. Excessive activation of systemic inflammatory cascades, mediated by pro-inflammatory cytokines and membrane receptor signaling, as well as microbiota dysregulation and hypoxic injury of the premature intestine due to micro-thrombotic events are three recognized mechanisms in constant interplay [1,9,16,17]. Diagnosis based on clinical and radiographic findings is often challenging and established in later disease stages [15]. Given NEC’s subtle onset, predicting its progression is crucial for optimizing patient outcomes. In this context, several potential biomarkers in serum, urine and stool samples have been evaluated [18,19,20,21,22]. However, identifying a single molecule that combines features such as broad availability, rapid and timely increase and decrease, satisfactory ability to differentiate NEC from similar clinical entities, non-invasiveness and cost-efficiency, has been challenging [20,23,24,25]. Instead, serial measurement of non-specific parameters, such as C-reactive protein (CRP) and procalcitonin (PCT) are commonly used in clinical practice for NEC diagnosis and treatment monitoring [20,21].

Complete blood count (CBC) is the most common laboratory test performed and can provide information about all blood cell components and their respective indices, allowing timely recognition of pathologic patterns and evaluation of clinical findings [23]. CBC has been increasingly acknowledged due to the advantages it presents, such as being a low-cost exam with a fast turn-around time, accessible in all health-care facilities. Hematologic parameters interpretation in newborns, especially preterm infants, should take into account the physiologic changes after birth, as values are expressed in age-specific intervals rather than standard reference ranges. Unlike adults, neonatal CBC values vary depending on both gestational age and postnatal age. These parameters undergo dynamic changes, including shifts in erythropoiesis, leukocyte distribution, and platelet homeostasis. Such variations can complicate the interpretation of CBC results, particularly in distinguishing between pathological changes and normal developmental fluctuations [24,25]. Many CBC parameters reflect tissue inflammation and damage in preterm neonates. Since NEC has an inflammatory substrate, it causes several CBC alterations, even before symptom manifestation. The hematologic abnormalities in neonates diagnosed with NEC, were first described by Hutter et al., who reported an increased incidence of thrombocytopenia, diffuse intravascular coagulation (DIC), abnormal absolute neutrophil count and hemolytic anemia in these patients [26]. Even though most cases are usually mild with no need of intervention, certain patients with severe NEC require blood product transfusion and increased vigilance for short and long-term complications [26,27,28]. Given NEC’s inflammatory substrate, it induces CBC alterations even before clinical symptoms become evident. Several hematologic indicators, including thrombocytopenia, neutrophil-to-lymphocyte ratio, and immature-to-total neutrophil ratio, have been investigated for their potential value in NEC detection and prognosis [29,30,31]. While some studies have incorporated CBC parameters into NEC severity scoring models [32,33], their application in routine clinical practice remains limited primarily due to variability in findings and a lack of standardized cut-off values.

In this systematic review, we aim to summarize the literature on studies examining the diagnostic utility of distinct CBC parameters for NEC diagnosis, as well as their association with various clinical outcomes.

## 2. Materials and Methods

### 2.1. Search Protocol/Databases

We conducted a systematic review to excerpt, assess and interpret published studies addressing our research goal. A protocol was developed for this systematic review, according to the Preferred Reported Items for Systematic Reviews and Meta-analysis guidelines (PRISMA; presented as a Supplementary Materials) [34], registered in the PROSPERO database (CRD42024517534).

Our research objective was to determine the diagnostic and prognostic potential of various CBC parameters in neonates with NEC. Randomized controlled studies (RCT) and observational studies focusing on the NEC population were included. We focused on studies in which NEC diagnosis was based on standardized diagnosing criteria, on Bell’s criteria, “2 out of 3 rule”, or/and any standardized criteria for the diagnosis of NEC [35]. Additionally, the CBC parameter(s) reviewed were clearly defined. All review articles, including systematic reviews, as well as meta-analyses and conference proceedings were excluded from the present study. Articles published in languages other than English were excluded from the review. Additionally, unpublished studies and preprints were not included as part of the study selection process.

A systematic review of the literature in the Pub Med and Scopus databases was conducted, between February 25 and December 2024. A combination of the following keywords was used in the search algorithm: Premature, Neonate*, Preterm*, SGA, “Small-for-Gestational-Age”, “Intrauterine Growth Restriction”, “IUGR neonates”, “Very low birth weight”, VLBW, Infants, “necrotizing enterocolitis”, NEC, Platelets, PLT, “mean platelet volume”, MPV, “platelet distribution width”, “PDW”, Plateletcrit, “Platelet Indices”, “red blood cells”, RBC, “Nucleated red blood cells”, NRBC, “Neutrophils”, Hematocrit, Hemoglobin, Leukocytes, Macrophages, Neutropenia, Neutrophilia, “immature to total neutrophil ratio”, “I/T ratio”, “platelet to lymphocyte ratio”, “Eosinophils”, “PLR ratio”, “Thrombocytopenia”, “Absolute Monocyte count”, “Absolute Lymphocyte count”, “complete blood count”, linked by Boolean logical operators (AND, OR). For the purpose of minimizing the risk of missing relative studies, a manual search was conducted by reviewing references from all selected studies and other systematic reviews with similar research topics.

### 2.2. Study Outcome(s)

Diagnosis of NEC

NEC development

Need for surgical or medical management

Mortality

### 2.3. Data Synthesis and Presentation

The following data from each study were recorded in a data extraction form: first author, country of origin, year of publication, hematologic parameter(s) studied and their respective cut-off values, clinical and demographic characteristics [such as birth weight (BW), GA and sex] and study endpoint (i.e., mortality, need of surgical or medical management). We also recorded, when available, the reported performance measures of each CBC parameter, either alone or as part of a scoring system, in predicting certain clinical outcomes in neonates with NEC.

### 2.4. Disagreement Resolution

Data were independently extracted and their quality assessed by three authors (P.M., A.G.T., A.E.P.). In cases of dispute between the two researchers, resolution was achieved after discussion or with the contribution of a third researcher (RS), when necessary.

## 3. Results

**Figure 1:**
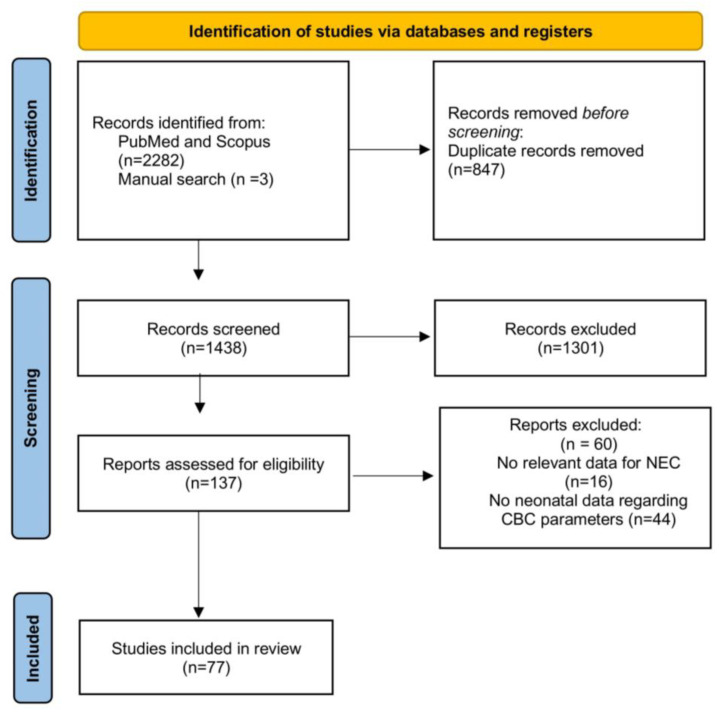
Flowchart of Study Selection Process.

Our initial search in the Pub Med and Scopus databases yielded a total of 2285 studies, of which 847 were duplicates and subsequently removed using the default settings of the reference managing tool EndNote X8 (Clarivate, Philadelphia, PA, USA). After carefully screening the remaining abstracts, an additional 1301 were excluded, either due to meeting at least one of the exclusion criteria or their irrelevance to our research question. A detailed full-text examination of the remaining 137 studies determined that only 77 [29,30,31,32,33,36,37,38,39,40,41,42,43,44,45,46,47,48,49,50,51,52,53,54,55,56,57,58,59,60,61,62,63,64,65,66,67,68,69,70,71,72,73,74,75,76,77,78,79,80,81,82,83,84,85,86,87,88,89,90,91,92,93,94,95,96,97,98,99,100,101,102,103,104,105,106,107] met all the inclusion criteria and were therefore included in this review. The selection process is illustrated in Figure 1. Out of the 77 included studies, 6 were prospective, while the remaining 71 were retrospective. The characteristics of each study are presented in Supplementary Materials (Table S1). Data from a total of 295,195 neonates were analyzed, of whom 14,570 had an established NEC diagnosis.

## 4. NEC-Associated Mortality

Although advancements in neonatal intensive care have improved the overall outcomes, NEC continues to be a major cause of surgical intervention, postoperative complications, and mortality among premature infants. Out of the 77 studies, 17 focused on NEC-associated mortality as a primary outcome (Table 1).

Several studies identified thrombocytopenia as a critical predictor of NEC severity and mortality [63,89,99,108]. Kenton et al. [100] found that severe thrombocytopenia within the first 3 days of NEC diagnosis was strongly associated with adverse outcomes, including bowel gangrene and increased mortality. Ragazzi et al. [59] demonstrated that both platelet count and platelet-neutrophil (PN) product were lower in non-survivors, although PLT count alone remained a strong predictor of mortality. Al Tawil et al. [99] reported that thrombocytopenia significantly increased the risk of death (OR = 33.6), while Atici et al. [63] determined an optimal cut-off PLT level of 110,000/µL for mortality prediction. Similarly, Zouari et al. [89] found severe thrombocytopenia to be a key predictor of NEC-related death.

Neutrophil-related parameters were also linked to NEC severity and outcomes. Gordon et al. [103] found that non-survivors had higher total WBC, absolute neutrophil counts, and segments/bands, while Kordazs et al. [97] noted that an immature neutrophil proportion above 34% at NEC onset correlated with stage III NEC, and a WBC count > 22 G/L was linked to severe disease. Qin et al. [44] identified that a drop in absolute neutrophil count (ΔANC) was the most sensitive predictor of severe NEC and death, especially when combined with platelet count.

Anemia was another key factor in NEC prognosis. Gordon et al. [103] reported that neonates who died had lower hemoglobin and hematocrit levels. Similarly, Kordazs et al. [97] linked low Hb levels to severe NEC and mortality. Feng et al. found that lower Hb and WBC levels were independent predictors of NEC surgery and death. Assenga and Tooke [79] further emphasized that anemia requiring RBC transfusion significantly increased mortality risk in NEC cases.

Across these studies, thrombocytopenia emerged as the most consistent CBC predictor of NEC mortality, with low platelet counts strongly linked to poor outcomes. Neutrophil fluctuations and anemia further enhanced prognostic accuracy. These findings highlight the importance of hematologic monitoring in NEC management and early intervention strategies.

## 5. Anemia

Anemia is a common condition among premature infants upon admission to the Neonatal Intensive Care Unit (NICU), as they represent a distinct population within transfusion medicine. In preterm and critically ill neonates, anemia arises from a complex interplay of factors, including perinatal stress, early cord clamping, inadequate iron stores, inflammatory responses, all of which can impair the infant’s ability to sustain optimal hemoglobin (Hb) levels for effective tissue oxygenation. In term neonates, the “physiologic Hb nadir” occurs at 8–12 weeks postnatal, approximately 11 mg/dL, at which point erythropoietin (EPO) production is stimulated. In contrast, anemia in preterm infants results in more profound deficits compared to physiological anemia, primarily due to hematopoietic immaturity, erythropoietin deficiency, and deficient iron transport across the placental circulation iatrogenic blood losses, rapid growth rates, and underlying comorbidities. The Hb nadir is lower in preterm neonates, and erythropoietin production is stimulated at even lower Hb levels (7–9 g/dL) [109,110]. This recognized pathologic entity, defined as “anemia of prematurity” (AOP), is more prominent in small for GA (SGA) and very low birth weight infants (VLBW), leading to clinical abnormalities and increased transfusion rates in these patients [111,112].

Clinically, anemia becomes significant when oxygen-carrying capacity is compromised, leading to inadequate tissue perfusion and metabolic dysfunction. Lower Hb values translate into impaired tissue oxygenation and increased anaerobic metabolism and oxidative stress, resulting in tissue damage due to release of reactive oxygen species (ROS) and lactate, which is a negative prognostic index in itself [113]. Anemia also impairs the usual changes in vascular resistance following delivery. The main pathophysiologic mechanism involves inadequate EPO production by the liver (the primary organ responsible for EPO production during fetal life), after transition to the less hypoxic extra-uterine environment [109]. In preterm neonates with NEC, reduced perfusion of the immature gut renders it more susceptible to mucosal injury, activation of inflammatory cytokines and epithelial cell necrosis. The relationship between NEC and anemia seems to be bidirectional, since NEC may in its own turn worsen the pre-existing anemia through GI blood loss or enhancing coagulopathy and intravascular hemolysis in the presence of DIC. Systematic inflammation also suppresses EPO-mediated signaling, resulting in impaired erythropoiesis [110].

In recent years, concerns have emerged regarding the potential association between red blood cell (RBC) transfusions and the development of NEC, resulting in the establishment of more restrictive transfusion criteria for premature infants. However, the evidence regarding the causal link between anemia, RBC transfusions, and NEC remains conflicting. The terms “transfusion-associated necrotizing enterocolitis” (TANEC) and “transfusion-associated acute intestinal injury” (TRAGI) are used to describe a range of serious gastrointestinal complications that occur in neonates following blood transfusion. While there is no universally accepted definition of TANEC, most studies characterize it as the onset of NEC within 48 h of RBC transfusion [114,115]. However, since most neonates receiving transfusions are already anemic, it is challenging to determine whether NEC results from the transfusion itself or the pre-existing anemia. The difficulty in distinguishing the effects of RBC transfusion from those of the underlying anemia that resulted in the transfusion complicates this issue. Moreover, animal studies have demonstrated that anemia can trigger an inflammatory response in the gut, mediated by macrophages, which contributes to intestinal injury. This inflammatory response may be exacerbated by subsequent RBC transfusions, leading to a further increase in inflammation and gut damage [115].

Multiple studies have attempted to define the association of anemia with NEC development and severity (Table 2).

Observational studies indirectly support the role of pre-transfusion anemia in heightening the risk of NEC. In a study by Song et al., 467 neonates with different birth weights (<1500 g, 1500–2499 g, ≥2500 g) who developed NEC stage II or III were included, with eligible controls matched 1:1 for VLBW infants based on gestational age, sex, and birth weight. Subgroup analysis pointed that severe anemia within 72 h of birth was a significant risk factor for NEC development (OR = 2.404, *p* = 0.016) in VLBW infants but did not affect NEC severity [101]. Anemia was also reported to increase the risk of NEC in VLBW infants in a case-control study by Singh et al., which demonstrated that a one -point decrease in hematocrit (Hct) nadir resulted in a 10% increase in risk of early onset NEC (manifested within the first 3 weeks) [43]. In the same study, iron supplementation was found to act as a protective factor against NEC.

Patel et al. [46] conducted a multicenter prospective study, enrolling 598 VLBW infants admitted in the NICU within a 4-year period; 44/598 neonates (7.4%) developed NEC. The authors observed a 6-fold increase in NEC incidence in VLBW infants with Hb < 8 g/dL in a given week and suggested that severe anemia, rather than RBC transfusion might be the deciding factor for NEC development. In a 2020 retrospective study, both moderate (decrease < 15 g/L) and severe (decrease > 15 g/L) decrease in Hb concentration compared to the baseline emerged as independent risk factors for NEC development in preterm neonates with late onset sepsis [36,116].

Decreased Hb and Hct values have been associated with rapid disease course, surgical NEC and poor prognosis in some studies [74,103]. In a retrospective study spanning over 10 years, Lin et al. [51] compared the clinical and laboratory parameters of neonates with fulminant NEC (FNEC—defined by need of surgical intervention or death within 48 h) with the respective parameters of neonates without FNEC. In this study, Hct was used as a categorical variable, with a cut-off value of Hct = 22%, and not a quantitative variable. Therefore, this should be considered when interpreting the lack of a statistically significant difference between the two groups. Recently, Song et al. [65] proposed a novel metaheuristic algorithm to predict NEC diagnosis and prognosis. They developed a feature selection and classification algorithm using pre-disease data for diagnostic classification and NEC risk prediction. Neutrophil percentage, breast milk feeding, probiotics, MCH, and anemia-RBC transfusion were identified as key predictors for classic and surgical NEC, playing a significant role in early diagnosis and risk assessment.

Enhancing the understanding of how RBC transfusions and anemia contribute to the development of NEC is essential, as more than 50% of very low-birth-weight (VLBW, ≤1500 g) infants receive at least one transfusion during their hospitalization [117,118]. It is important to note that previous studies investigating the relationships between transfusion, anemia, and NEC may have been constrained by small sample sizes, case-control designs, or a lack of evaluation of time-dependent exposures. Therefore, there is a critical need for researchers to prioritize conducting RCTs or large multicenter prospective studies that can systematically and consistently examine each RBC transfusion, anemia episode, and NEC outcome.

## 6. White Blood Cells (WBC)

White blood cells (WBC), including neutrophils, eosinophils, basophils, monocytes, and lymphocytes, each serve distinct functions in the immune response and contribute specific proportions to the overall WBC count. In the context of NEC, WBC levels can offer valuable information regarding the pathogenesis of systemic infection [119]. However, they are not consistently reliable indicators of the degree of intestinal epithelial damage and necrosis [120]. Several studies have investigated the diagnostic and prognostic significance of WBC count and specific types of WBCs in neonates with NEC, highlighting their potential role in understanding disease progression and outcomes.

### 6.1. Neutrophils and Relative Indices

Neutrophils or polymorphonuclears (PMN) comprise 50–60% of the circulating leukocytes and play a critical role as the primary defensive cells to be recruited during the inflammatory response. PMN terminate external pathogens through a variety of potent mechanisms, including release of toxic antimicrobial substances contained in their granules and macrophage chemotaxis to enhance phagocytosis [121]. PMN are stimulated by bacterial products (pathogen-associated molecular patterns—PAMPS) and substances released after cell injury (Damage-associated molecular patterns—DAMPs) [122]. Afterwards, they migrate across the intestinal epithelium into the lamina propria with the help of membrane adhesion molecules in order to marginalize the infection site before their programmed apoptosis [123,124]. The migration process is enhanced by epithelial hypoxia.

Neutrophilia and neutropenia in neonates are determined according to the absolute neutrophil count (ANC), just as in adults. However, in this population, the variety of definitions and reference ranges used, explains the discrepancies in the reported incidence of PMN disorders among studies [61,125,126,127]. A common definition describes neutropenia as ANC less than 2 standard deviations below the mean value or below the 5th percentile for a specific postnatal age [128,129]. Severe neutropenia is usually defined as ANC < 500 and persistent neutropenia as low ANC count for more than 5–7 days [38,130,131].

While inflammatory conditions such as NEC are typically accompanied by neutrophilia and a left shift, neutropenia is recognized as a negative prognostic marker in most studies [61,71]. Patel et al. reported increased incidence of neutropenia in neonates who died of NEC compared to survivors [132,133], a finding further supported by other studies [50,63]. In their retrospective study spanning over 10 years and including 7300 infants, Christensen et al. noted a fourfold increase in the odds of NEC development in neutropenic SGA neonates compared to GA matched controls [52]. Early evaluation of neutropenia may contribute to predicting the extent and severity of the disease in a timely manner, as well as its progression to more advanced stages.

Recent cohorts have consistently described a reduced ANC on the day of NEC onset as a predictor of adverse outcomes, such as rapid disease progression to FNEC (defined as the need for surgical intervention and death within 48 h), even after multivariable regression analysis [51,72,82,96]. In these studies, neutropenia was often accompanied by other hematologic abnormalities. A drop in ANC (combined with thrombocytopenia) within 48 h of NEC onset has also been associated with negative prognosis, due to severe surgical NEC or FNEC [44]. On the contrary, ANC elevation by 5% indicated reduced risk of FNEC, according to a predictive model by Garg et al. [52]. Interestingly, a recent study by Dantes et al. [53] found no statistically significant impact of CBC parameters in the differential diagnosis between spontaneous intestinal perforation (SIP) and surgical NEC in VLBW neonates. Hence CBC elements were not included in their NEC predictive model.

ANC is not the only PMN parameter valuable for NEC patient evaluation. The immature to total PMN ratio (I:T ratio), gradually decreases from 0.16 to 0.13 within the first 60 h after birth and typically stabilizes at 0.12 for the first month of life [122,124]. In neonates with GA > 34 weeks, elevated I:T ratio and increased levels of immature PMN are reliable indicators of perforation and surgical NEC [42]. The correlation between the I:T ratio and disease severity, is even less well established in more preterm neonates [64,74]. Moreover, an I:T ratio > 0.5 was identified as a prognostic factor for death related to FNEC in a case-control study by Lambert et al. [57] and has been included in a proposed scoring system (along with other laboratory parameters) to swiftly predict the need for surgical intervention [81].

Increased neutrophil to lymphocyte ratio (NLR) reflects immune system dysregulation in cases of worsening systemic inflammation, infection or stress and has been associated with negative outcomes in various studies [123,132,134,135,136]. This finding is not only attributed to PMN elevation, but also to increased apoptosis of peripheral lymphocytes [136]. In the neonate population, NLR has played a significant role in predicting sepsis or NEC in comparison to traditional biomarkers such as WBC, CRP and absolute cell counts [47,130]. NLR ratio has also shown a positive correlation with the stage of NEC [56]. NLR may also be affected by other co-morbidities, such as congenital heart defects [137]. While NLR values of ≤1.60 and >3.20 have been reported to correlate with a reduced risk of NEC, universally accepted cut-off values for NLR in NEC diagnosis and prognosis have yet to be established [45].

Neutropenia in NEC patients has been attributed to PMN infiltration of the intestinal wall and peritoneum, as well as increased attachment to the microvascular wall around the sites of inflammation [59,71,138]. Autopsies have revealed normocellular bone marrow, supporting the aforementioned hypotheses [139]. Recombinant granulocyte-colony stimulating factor (rG-CSF) and recombinant granulocyte-macrophage colony stimulating factor (rGM-CSF) administration for the treatment of neonatal neutropenia has been used with varying efficacy, with most data concerning neonates with early onset sepsis [133,140,141]. In the meta-analysis by Bernstein et al., similar benefits were noted in neonates with septicemia treated with rG-CSF, but statistical significance was lost after exclusion of non-randomized studies [142]. A meta-analysis of 13 RCTs reported significant reduction of sepsis-related mortality in preterm, LBW infants that were treated with Rg-cSF, without effecting sepsis complications, including NEC [143]. Overall, hematopoietic colony-stimulating factors are not routinely administered in neutropenic neonates, including cases of NEC; none the less their use is individualized. However, in an animal model, rG-CSF demonstrated their protecting effects against hypoxia-induced intestinal damage [144]. Future studies are warranted to optimize their use in neonates with systematic infections, with further emphasis on the field of NEC.

### 6.2. Lymphocytes

Lymphocytes are essential regulatory components of the immune response and are massively recruited in the premature intestine of neonates with NEC, a phenomenon mediated by toll-like receptor 4 (TLR-4) signaling [145]. Lymphopenia, commonly defined as absolute lymphocyte count (ALC) < 3000/mL, often indicates immunodeficiency [146,147,148] and predisposes to more severe NEC phenotypes and NEC-associated sepsis [52]. Lymphopenia has been suggested as a potential predictor for the need for surgical intervention in neonates with NEC [30,87,96], while a one-unit increase in ALC within 24 h has been shown to have a protective effect against the progression to surgical NEC [72].

In the multi-center study by Lambert et al., 532 neonates with NEC were enrolled. Among these, 35 out of 532 cases (6.7%) progressed rapidly in a fulminant fashion, resulting in death within 48 h [66]. Upon analyzing the results, the researchers observed a correlation between lymphopenia within 72 h before symptom onset and the progression to FNEC as well as increased mortality. Li et al. [82] reported similar findings, highlighting that lymphopenia presenting within 24 h of NEC onset was linked to an increased risk of fulminant NEC as well as rapidly progressive necrotizing enterocolitis (RP-NEC) [85,96].

Hematologic response patterns after NEC onset may vary depending on GA. In their study, Garg et al. [52] included 184 infants with NEC, dividing them into three subgroups based on GA. Specifically, 130 neonates were placed in Group A (GA < 28 weeks), 30 neonates in Group B (GA 28–32 weeks), and 21 in Group C (GA > 32 weeks). The neonates were evaluated with CBC at daily intervals following the development of NEC. In Group A, the neonates who died from NEC had lower absolute lymphocyte counts (ALC) at 72 and 96 h compared to survivors, and in comparison, to the other GA groups. In the older GA groups, ALC levels were consistently lower (at 0, 24, 48, 72, and 96 h) in infants with surgical NEC compared to those with medical NEC. Conversely, an increase in ALC was linked to a decreased need for surgery in Group A neonates. In another retrospective study, neonates with FNEC and surgical NEC had significantly lower ALC at day 2 and 7 after disease onset, respectively, compared to patients who recovered conservatively [72].

Frequent CBC measurements should be conducted to monitor ALC after the diagnosis of NEC, as a decrease may signal progression to surgical NEC, particularly in neonates with a GA of less than 33 weeks. These evaluations are essential for early identification of worsening conditions and to guide decisions regarding surgical intervention [67]. Interestingly, ALC might not fully recover in follow-up measurements, even after the completion of antibiotic treatment, a pattern not observed in other blood cell types [67]. This statement particularly pertains to preterm neonates with surgical NEC.

Since other researchers have observed higher mortality rates in NEC patients with elevated lymphocyte counts at the time of diagnosis [75,96], the results on the prognostic implications of ALC changes in NEC patients have been inconsistent. Following multivariate analysis, Gordon et al. [103] hypothesized that the included neonatal population’s GA may produce a dichotomous dynamic, which would account for the disparities in study findings. Specifically, a higher ALC was associated with worse prognosis in neonates with a higher GA, but it was linked to an increased likelihood of survival in more preterm infants. This suggests that the role of ALC as a prognostic factor may differ based on gestational age, highlighting the importance of tailoring diagnostic and treatment strategies to the specific needs of preterm neonates [67,72,103]. Peripheral lymphocyte percentage did not differ between patients with mild (Stage I and Stage IIa) and severe NEC, according to another study [110]. Another retrospective study found no statistical difference in ALC at birth between NEC subjects and controls, however small sample size may have affected these results [83]. Correlation between lymphocyte counts and disease severity should be addressed in future studies. Finally, other indices involving ALC, such as NLR and PLT: lymphocyte ratio (PLR) is discussed in the “Neutrophils” and “Platelets” sections, respectively.

### 6.3. Eosinophils

Eosinophils normally comprise a small minority (up to 3%) of circulating blood cells and exhibit homeostatic, proinflammatory and immunomodulatory properties, mainly in cases of allergic reaction, parasitic infection, autoimmune disease or malignant conditions [146,149,150,151]. Their action is effectuated through secretion of eosinophil-specific cytotoxic substances contained in their granules, phagocytosis, release of chemoattractant molecules and antigen presentation to T-helper cells [72,146,147].

Eosinophilia in neonates is defined as an absolute eosinophil count (AEC) ≥ 700 cells/μL, while in severe eosinophilia AEC exceeds 3000 cells/μL [148]. Even though eosinophil infiltration of the GI wall characterizes multiple disorders that effect different locations of the GI tract in adults, from the esophagus to the colon, their role in NEC pathophysiology is still elusive [149]. Eosinophils possibly participate as mediators in both the initial inflammatory process and tissue repair after injury in NEC subjects [150]. Eosinophilia is commonly observed within 2–3 weeks of life, when the newborn organism enters an anabolic state [125]. This hematologic alteration is more frequently observed in hospitalized preterm neonates with a GA of less than 30 weeks. It often correlates temporally with factors such as transfusion, infection, and NEC [147].

Christensen et al. [152] conducted a retrospective study involving 271 neonates who presented with bloody stools, aiming to evaluate the value of AEC in distinguishing between NEC and allergic enteropathy, a less recognized and typically more benign condition with lower mortality in the NICU [153]. Of the 271 patients, 54 had AEC levels above the 95th percentile for their gestational age and postnatal age. The analysis revealed that eosinophilia was more common in neonates who had undergone transfusion within the previous 48 h. Neonates with a prior history of blood transfusion demonstrated a progressive increase in eosinophil counts. In contrast, those who developed bloody stools and eosinophilia but did not have a history of transfusion, experienced a decrease in eosinophil counts after feeding was discontinued and generally had a more benign disease course. While the exact mechanism behind transfusion-associated eosinophilia remains unclear, it is hypothesized that the exposure to foreign antigens present on donor red blood cells (RBCs) or medications administered during the transfusion, may contribute to the observed eosinophilic response. However, there were no significant differences in outcomes, such as enteric wall pneumatosis, need for surgery, or mortality due to NEC, between the eosinophilic neonates and those with AEC within the reference range. This suggests that while elevated AEC might be indicative of certain clinical factors, it does not necessarily correlate with more severe outcomes in neonates with NEC. In a small cohort study by Patel et al. [46], eosinophilia was consistently observed in all cases of NEC, regardless of GA. This finding suggests that the increase in AEC may be a response to gastrointestinal (GI) microbiota dysregulation, a factor often associated with the pathogenesis of NEC. The study implies that eosinophilia could potentially serve as an indicator of microbiota-related alterations in the gut, which may contribute to the development of NEC in neonates.

Wahidi et al. [60] used a Likert scale to explore the prognostic significance of eosinophilia for surgical complications associated with NEC, including persistent feeding deficiencies due to strictures. The researchers concluded that early persistent eosinophilia (defined as ≥5% of the total leukocyte count for ≥5 consecutive days following the onset of NEC) was a risk factor for all studied adverse outcomes such as intestinal strictures and/or liver fibrosis. However, peripheral eosinophilia was not associated with elevated eosinophil concentrations in the stool or signs of eosinophilic colitis. Interestingly, neonates in the eosinophilic group had lower baseline BW and GA compared to both the control group and those with NEC without persistent eosinophilia. These two factors—lower BW and GA—could potentially confound the relationship between eosinophilia and the observed adverse outcomes. Eosinophil activation is usually preceded by production of interleukins IL-13 and IL-33 by the damaged gut epithelium [154]. These cytokines are subsequently involved in promoting tissue fibrogenesis and stricture formation [155].

### 6.4. Monocytes and Relative Indices

Interest in the role of monocytes in NEC immunopathology has emerged since the revelation of a macrophage-rich infiltrate in histologic samples of resected bowel, differentiating it from adult GI disorders [156]. Macrophage activation is excessive in NEC patients, partly due to reduced concentrations of the down-regulating tumor growth factor-β (TGF-β) molecule [17,157]. Constant monocyte accumulation and differentiation in the lamina propria reduces the number of circulating monocytes while the pool of bone marrow monocytes is limited in preterm neonates [61,154]. Monocytopenia or a decrease in absolute monocyte count (AMC) from baseline is commonly observed in IUGR neonates; however, it is rarely an isolated finding. In contrast, isolated monocytopenia appears to be a distinctive hematologic feature of NEC, potentially serving as a useful marker for early disease identification in affected neonates [38]. As a result, AMC has been viewed as an appealing biomarker for NEC diagnosis and prognosis.

Tajalli et al. [77] reported reduced AMC in NEC neonates compared to controls, as well as reduced AMC in Stage III vs. Stage II patients, with ROC analysis showing a diagnostic accuracy (AUC-area under the curve) of 0.693 (95% confidence interval [CI]: 0.612–0.773) and 0.738 (95% CI: 0.627–0.850), respectively.

A reduction in AMC from the last asymptomatic measurement can distinguish Stage II and III NEC from other disorders causing feeding intolerance in VLBW infants, with an NPV of 88% [27]. Desiraju et al. [105] enrolled 283 infants in their retrospective study, analyzing 4 subgroups: rule-out NEC, stage II NEC, stage III NEC and isolated bacteremia confirmed by positive blood culture. Baseline characteristics were similar among groups. Analysis suggested that a 75% reduction of AMC from pre-symptomatic AMC (measured a median of 3 days prior to illness onset) is indicative of Stage III NEC development and can distinguish it from Stage II, while the absence of an AMC drop in bacteremic neonates safely excludes the presence of NEC.

Significant AMC drop may also contribute in distinguishing surgical NEC from medical NEC in infants with GA < 33 weeks. In a study by Pantalone et al. [67], the observed AMC reduction from baseline was smaller than previously reported, possibly due to NEC occurring at earlier postnatal ages. During this period, monocyte populations undergo rapid shifts influenced by environmental factors, which may account for the variation in findings across different cohorts [158]. Finally, Gordon et al. [103] reported decreased AMC and monocyte percentages in neonates who died of NEC compared to survivors. Moroze et al. [49], investigated the trends of blood AMC over 72 h in 130 neonates with suspected NEC and found that a decrease in AMC can serve as an adjunct biomarker to help confirm the NEC diagnosis and differentiate between its stages.

## 7. Nucleated Red Blood Cells

Nucleated red blood cells (nRBCs) are immature forms of erythrocytes typically found in the bone marrow, where they are produced during erythropoiesis. These cells eventually mature into reticulocytes and, ultimately, mature erythrocytes. Physiologically, their presence in peripheral circulation, which is normal in fetuses, ceases within the first month after birth [159,160]. Elevated nRBCs are usually a result of the stress induced by chronic or subacute hypoxia (including intrauterine hypoxia), hemolysis, inflammation or trauma [161,162]. nRBC increase is associated with extramedullary hemopoiesis, increased mortality and various negative outcomes in critically-ill adults and children [160,161,163,164,165,166,167].

Mandel et al. [83] sought to explore the use of nRBC count in NEC. Τo avoid confounding, the authors excluded neonates exposed to known nRBC increasing factors, such as pregnancy-induced hypertension, maternal gestational diabetes, perinatal infection, chromosomic abnormalities and SGA status, eventually including a total of 46 infants (23 with confirmed NEC and 23 controls) in their study. The authors reported that nRBC count at birth was the only parameter significantly different between neonates that eventually developed NEC and control infants, a fact possibly attributed to exposure to conditions of intrauterine hypoxia. Backwards logistic regression identified NEC as the independent factor predicting nRBC elevation. Furthermore, Baschat et al. [168] conducted a retrospective analysis of nRBC parameters in 176 very preterm neonates with intrauterine growth restriction (IUGR), investigating NEC as one of the studied outcomes. Their findings indicated that nRBC counts exceeding 30 per 100 white blood cells and persisting beyond three days served as a valuable prognostic marker for NEC development in this high-risk population.

## 8. Platelets and Platelets Indices 

### 8.1. Thrombocytopenia

Thrombocytopenia is a significant and common complication in neonates with NEC. This serious GI condition often leads to a marked decrease in platelet count. Research indicates that between 50–95% of neonates with NEC develop thrombocytopenia, defined as a platelet (PLT) count of less than 150,000/μL, within 24–72 h of the disease diagnosis [71,169]. This drop in PLT count can serve as an important clinical indicator of disease deterioration. Furthermore, in more severe NEC cases where surgical intervention is necessary, the incidence of thrombocytopenia is even more pronounced. In these neonates, PLT counts often fall below 60,000/μL [128,170]. The significant drop in PLT count in surgical NEC patients underscores the critical nature of the disease and the increased risk of complications, including bleeding and infection [79,169,171]. PLT transfusion is often utilized in the management of neonates with NEC, particularly in cases where thrombocytopenia is severe or associated with active bleeding. However, the use of PLT transfusions in this context is a subject of debate due to potential risks and uncertain benefits [171,172,173,174].

NEC-associated thrombocytopenia can arise from various mechanisms, reflecting the complex pathophysiology of this condition. The primary mechanism implicated in thrombocytopenia associated with NEC is the peripheral aggregation and destruction of PLTs in the microvasculature [71,175]. This process reflects the localized inflammatory response and endothelial dysfunction characteristic of NEC, leading to impaired PLT adhesion and increased clearance, contributing to decreased PLT counts. Overall, neonates with NEC exhibit a hypercoagulable profile, as evidenced by the assessment of rotational thromboelastometry (ROTEM) parameters [176]. In patients diagnosed with NEC, there is often a rapid decline in platelet counts, with transfusions providing only temporary and limited improvements. In animal models, PLT activation is mediated by thrombin and is regarded as an early pathogenetic event during the course of intestinal injury [175]. Furthermore, preclinical investigations have shown that targeted inhibition of thrombin using a nanomedicine-based approach was observed to have a protective effect in patients with NEC, without an increase in the risk of bleeding events [175]. The exact location of platelet consumption remains uncertain, although the occurrence of microthrombotic events within the affected intestine is regarded as a significant possibility. From a conceptual standpoint, various agents such as platelet-activating factor (PAF), arachidonic acid metabolites, coagulation factors and bacterial products are considered to play a key role in the pathogenesis of NEC [176,177,178]. These agents can activate endothelial cells and macrophages, particularly in conjunction with thromboplastin released from the necrotic bowel tissue, thereby triggering the release of secondary mediators like inflammatory cytokines and nitric oxide [179]. Collectively, the stimuli mentioned above contribute to platelet activation and aggregation within the microvasculature. The antimicrobial action of PLT is also triggered by interaction with the complement pathway [176]. The complex interlink between systemic inflammation and hemostatic imbalance has been investigated extensively in recent years [180,181].

NEC neonates exhibit lower mean PLT counts compared to healthy controls [58]. Whether thrombocytopenia is only a marker of poor outcomes or it directly influences these outcomes is still debated among researchers [182]. Regardless, most studies correlate thrombocytopenia with an increase in NEC mortality [59,99,102,103,108]. Kenton et al. [100] retrospectively reviewed the medical records of 91 preterm neonates with Stage II and III NEC. 47/91 patients had severe thrombocytopenia (PLT < 100,000/μL) within 72 h of NEC diagnosis and patients were stratified according to the presence or absence of this finding. Final analysis reported increased mortality and GI complications (i.e., cholestasis and short bowel syndrome) rates in the presence of severe thrombocytopenia (adjusted OR = 6.39; *p* = 0.002, and adjusted OR = 5.47; *p* = 0.006, respectively).

Thrombocytopenia might increase the risk for severe NEC forms, as well as NEC-associated sepsis [52,57]. Su et al. [119] demonstrated a negative correlation between NEC grade and PLT count in their cohort, which included 46 neonates with mild NEC (Stage I and Stage IIa) and 23 neonates with severe NEC. The authors suggested that PLT, combined with elevated serum intestinal fatty acid binding protein (I-FABP) and WBC count, could reflect the degree of intestinal injury. Similarly, Ververidis et al. [102] reported that a platelet counts of less than 100 × 10⁹/L or a rapid decline in platelet count represents a poor prognostic factor. In another multi-year study, Miner et al. [64] recognized significantly reduced PLT counts among neonates who deteriorated from Stage II to Stage III disease, in line with the results of Hällström et al. [74]. Neonates who die of NEC have also exhibited lower nadir PLT counts compared to survivors [63,102]. However, although thrombocytopenia is more common in patients with fulminant NEC than in neonates with milder forms of NEC, this relationship was not statistically significant in a 2019 study by Lin et al. [51]. These findings contrast with those of other cohorts, in which a decrease in platelet count was not predictive of progression to surgical NEC [32,106]. Based on the findings of the aforementioned studies, monitoring platelet count during the course of NEC is valuable; however, it should not be solely relied upon for prognostication, as its predictive value remains limited.

In a study assessing the use of coagulation parameters, Feng et al. [40] identified thrombocytopenia as an independent risk factor for surgical NEC. Among the tested parameters, activated partial thromboplastin time (APTT) demonstrated the best diagnostic performance based on AUC analysis. Notably, only 38.7% of all infants in the study exhibited coagulopathy. Gupta et al. [81] proposed a scoring system that incorporates specific laboratory parameters measured 4–12 h and 12–24 h after NEC diagnosis, aiming to accurately identify NEC patients who would require laparotomy. One point was awarded for each of the following parameters: WBC < 9000/mm^3^, I:T >0.5, PLT < 200,000/mm^3^, and BE < or = −2. While a score of ≥3 demonstrated good performance in predicting the need for surgical intervention, it should be noted that the cutoff value for thrombocytopenia is defined as <200,000 mm^3^, which means that some results today would be considered within the normal range, affecting the final score of certain neonates. Huang et al. [94] conducted a study aimed at exploring the clinical characteristics of NEC complicated by intestinal perforation and predicting the incidence of intestinal perforation in NEC cases. They reported that thrombocytopenia and hypoalbuminemia may serve as independent risk factors for predicting intestinal perforation in neonates with NEC. A nomogram model incorporating these factors demonstrated strong predictive accuracy for identifying NEC patients at risk of intestinal perforation.

Recognizing infants at high risk of post-NEC strictures is helpful in adjusting enteral re-feeding protocols, limiting total hospitalization time and mitigating stricture severity. In this regard, PLT counts have drawn researches’ attention. Gaudin et al. [80] conducted a retrospective study of 60 neonates with NEC ≥ Bell’s stage II, 14/60 receiving surgery in the acute disease phase and the rest treated with conservative measures. In both groups, stricture development was found to correlate with the presence of reduced PLT counts (<100.000 mm^3^), among other factors. Stricture incidence was increased in this study compared to the usual rates reported in literature, and this may be caused by systematic investigation with contrast study, a sensitive modality for stricture detection, even though some strictures caused no symptoms. Despite this factor, no deaths were attributed to stricture formation. A multi-center study by Zhang et al. [88], released 4 years later, did not report a statistically significant difference in mean or nadir PLT count between neonates that did and the ones that did not develop strictures after a 3-month follow up. It is worth noting that in this study, only infants suspected of having strictures underwent contrast enema to confirm and localize the stricture; contrast enema was not administered to asymptomatic patients. Interestingly, both studies reported statistically significant elevation in CRP levels [80,88]. Further investigation is required to expand upon these results.

Thrombocytopenia may not be present at the first CBC drawn, but increased vigilance is necessary should it develop later on. In the aforementioned study by Ververidis et al. [102], the association of PLT decline with intestinal gangrene was explored. In cases of necrosis, thromboplastin is released by the gangrenous bowel, catalyzing prothrombin conversion to thrombin and exacerbating clotting and PLT destruction. Rapid PLT count drop below 100,000/μL was a reliable predictor of bowel gangrene, similar to the findings of O’Neill et al. [102,181]. In these patients, PLT count did not recover to normal range within 24 h, a significant differentiation from septic neonates without bowel necrosis. According to other studies, a significant PLT decline within the first week or within the first month of life (>30% from baseline value) might assist in identifying infants with GA < 32 weeks at high risk for NEC development and infections, even in the absence of thrombocytopenia [57,92]. These findings highlight the need for constant PLT monitoring during hospitalization to enable timely detection of PLT decrease and appropriate intervention.

PLT derangements should be interpreted along with other CBC parameters, since multiple blood cell types are involved in the inflammatory process and PLT-leukocyte interaction is an integral component of the immune response [183]. In the study of Zheng et al. [30], PLR was defined as the mean ratio of PLT count to lymphocyte count in all blood tests within 1 week before NEC diagnosis. The authors measured the PLR in 200 neonates, including 93 NEC patients and 107 matched controls, finding significantly higher PLR values in the NEC group. A PLR > 100 was a reliable predictor of eventual NEC development in their adjusted model (OR = 18.82, 95% CI: 2.93–120.98, *p* = 0.002). According to a recent study conducted by Guo et al. [41], elevated PLR is associated with significant inflammation in patients with surgical or fatal NEC. The predictive model that integrates ANC, PLR, CRP, and PCT has the potential to differentiate between surgical or fatal NEC and medical NEC. This approach could enhance risk awareness and facilitate more effective communication between clinicians, ultimately improving patient management. However, additional multicenter research is necessary to confirm these findings and improve the clinical management of NEC. Chong et al. [68] conducted a study to examine gestational age-specific hematologic characteristics in preterm neonates with NEC and identify hematological biomarkers that can predict surgical NEC. Compared to medical NEC (m-NEC) at onset, surgical NEC (s-NEC) neonates exhibited distinct hematological patterns based on GA. In GA < 28 weeks, s-NEC infants had lower platelet counts. In GA 28–32 weeks, significant decreases in platelets, lymphocytes, and monocytes were observed. In GA 32–37 weeks, lower absolute lymphocyte counts were noted. Independent predictors effectively distinguished s-NEC from m-NEC, with AUC of 0.880 for platelet counts in GA < 28 weeks, 0.889 for C-reactive protein in GA 28–32 weeks, and 0.892 for lymphocyte counts in GA 32–37 weeks. Li et al. [93] developed a predictive model for rapidly progressive necrotizing enterocolitis (RP-NEC), a severe NEC subtype characterized by rapid progression and high mortality, using data from a retrospective cohort of 334 newborns with NEC. Six independent risk factors—low plasma sodium, elevated C-reactive protein, low platelet and lymphocyte counts, low blood pH, and ascites at NEC onset—were identified and incorporated into the model. The predictive model exhibited excellent performance, with an AUC of 0.983, highlighting its potential for early RP-NEC identification.

### 8.2. Platelet Indices

Finally, CBC provides certain adjunct PLT indices, namely mean PLT volume (MPV) and PLT mass index (PMI) and plateletcrit (PLCT). These parameters reflect PLT morphology, activation and turnover better than PLT count alone [88,184]. In cases of systemic inflammation and oxidative stress, the bone marrow attempts to compensate for PLT destruction by producing larger volume PLTs [185]. A disturbance of these indices in critically-ill adults is often driven by an excessive inflammatory process and is associated with a poor prognosis [88,186,187], while an increasing number of studies are being published examining their role in pediatric conditions [188,189,190,191].

Cekmez et al. [76] conducted a prospective study measuring the mean MPV at birth and at 72 h in 287 neonates with a BW < 1500 g and GA < 34 weeks. Of these, 21 neonates eventually developed NEC, and their laboratory results were compared to those of 152 controls. The study found significantly higher MPV values at both time points for neonates who developed NEC (*p* = 0.001 at birth and *p* = 0.0005 at 72 h). However, multivariate analysis was not performed in this study. Zhang et al. [29] examined the role of mean MPV and platelet distribution width (PDW) in predicting surgical neonatal NEC and explored their association with the severity and prognosis of NEC. The study results demonstrated that MPV, PDW, and fibrinogen (Fib) were independently associated with surgical NEC. Furthermore, the combination of MPV and PDW exhibited significant predictive value for surgical NEC. Elevated levels of MPV and PDW were correlated with the extent of intestinal necrosis and poor prognosis in surgical NEC patients. These findings may aid in prioritizing neonates for transfer to specialized neonatal surgical centers, optimizing surgical care, and providing essential guidance for parental counseling. Meng et al. [48] evaluated the predictive value of intestinal tissue oxygen saturation (rintSO_2_) combined with PCT and MPV in determining NEC severity in preterm infants. Their findings suggest that the combination of rintSO_2_, PCT, and MPV may serve as early biomarkers for NEC severity, facilitating early diagnosis and timely intervention to improve prognosis.

Hence, PLT count and PLT indices play a significant role in the diagnosis and outcome of neonates with NEC. Low PLT counts have been associated with an increased risk of NEC development, disease progression, and adverse outcomes such as bowel perforation and mortality. While PLT transfusions can help raise PLT counts and potentially reduce bleeding complications in NEC patients, there is also concern about the associated risks. PLT transfusions have been linked to increased morbidity in preterm infants, including risks of infection, transfusion reactions, and alterations in immune function [192]. Therefore, the decision to administer PLT transfusions in neonates with NEC should be made cautiously, weighing the potential benefits against the risks. Moreover, the prothrombotic nature of NEC underscores the importance of vigilant monitoring and management of coagulation parameters in affected neonates. Strategies aimed at mitigating thrombotic complications, such as anticoagulant therapy or targeted anti-inflammatory interventions, may hold promise in improving outcomes in this vulnerable population. PLT indices, including MPV, PDW, and PLCT, have also been investigated for their potential diagnostic and prognostic utility in NEC. Alterations in these indices may reflect changes in PLT function, activation, and turnover associated with NEC pathophysiology. Overall, monitoring PLT count and PLT indices in neonates with NEC can provide valuable information for early diagnosis, risk stratification, and management decisions. Close attention to these hematological parameters may help clinicians optimize care strategies for NEC neonates. Detailed data on studies assessing platelets and platelet indices are provided in Table S1.

## 9. Scoring System for Early Diagnosis and Prediction of NEC Severity in Neonates

Ongoing studies are focused on identifying a platelet threshold or other CBC parameter values that could predict the occurrence or prognosis of NEC. Neonates, especially preterm infants, are at high risk for developing NEC, making it one of the most common challenges encountered in NICUs. The fact that NEC is associated with increased mortality rates and negative impacts on both early and later outcomes highlights the importance of prioritizing its diagnosis and treatment for neonatologists.

However, two neonates with similar platelet counts or CBC parameter values, but at different clinical stages, may have very different risks for NEC associated morbidity and mortality. Therefore, it is crucial to predict which neonates are at risk of developing NEC and to quantify this risk using a model that incorporates not only CBC parameter values but also a range of clinical variables. Such a predictive model could help define indications for early intervention, serving as the first step towards personalized medical care for neonates.

In recent years, new clinical models have been developed and validated in various clinical scenarios to improve therapeutic approaches in adult patients [23,193]. However, data on NEC risk scales for diagnosis and prediction, as well as the prognosis of NEC progression in the neonatal population, remain limited. From our systematic review, we identified only 13 studies that have developed predictive models to assess the severity and prognosis of NEC in neonates by incorporating CBC parameters (Table 3).

These studies aimed to create scoring systems that integrate clinical, laboratory, and radiologic data to predict the risk of NEC progression, the need for surgical intervention, and the overall prognosis. McCormack et al. [62] aimed to identify clinical, radiologic, and laboratory factors to predict NEC severity, developing a score based on factors like enteral feeding time, blood pH, and WBC differential, with a score of ≥3 indicating high risk. Gupta et al. [73] focused on laboratory parameters measured after diagnosis to predict the need for surgical intervention, creating a scoring scale based on WBC count, platelet levels, and other factors. Kessler et al. [37] proposed a NEC score using lactate, gestational age, Bell stage, and platelet count to assess severity and survival outcomes. Li et al. identified neutropenia and other laboratory markers as key predictors for severe NEC and bowel resection [82,93]. Song et al. [65] introduced a metaheuristic algorithm using pre-disease data, focusing on key factors like neutrophil percentage and breast milk to predict NEC diagnosis and prognosis. Collectively, these studies emphasize the importance of combining clinical and laboratory data to predict outcomes and guide treatment strategies for neonates at risk of NEC.

## 10. Discussion

NEC remains one of the leading causes of morbidity and mortality in preterm infants, necessitating early diagnosis and effective intervention. Despite advances in neonatal care, NEC continues to have high complication rates, highlighting the critical need for reliable prognostic markers. As far as we know, this is the first systematic review analyzing hematological changes and their potential diagnostic and prognostic value in NEC. We hope that this addition will further contribute to the understanding and management of NEC in clinical practice.

The interpretation of hematological markers in NEC is influenced by physiological changes in neonatal hematology. Preterm infants have an immature hematopoietic system, reduced erythropoietin production, a predisposition to anemia due to low iron stores and frequent transfusion requirements [117,125]. Postnatal circulatory adaptation is accompanied by changes in white blood cell counts, with initial neutrophilia followed by subsequent neutropenia [125]. Differentiating pathological changes from physiological variations is essential for the accurate interpretation of CBC parameters in NEC. The results of studies included in this systematic review indicate that GA significantly influences the hematological response to NEC. Neonates with a lower gestational age (<28 weeks) exhibit more pronounced neutropenia and thrombocytopenia, while more mature preterm neonates (28–32 weeks) demonstrate progressive changes in their hematological profiles [68]. Understanding these differences is essential for tailoring diagnostic and therapeutic strategies based on gestational age.

Thrombocytopenia has been recognized as one of the most significant prognostic indicators of NEC, as studies have demonstrated an association between low platelet levels and increased mortality and disease severity [107]. The dynamic change in platelet count in NEC is crucial for the prognosis of the disease. Our findings suggest that a rapid drop in platelets within 24–72 h of symptom onset is associated with higher mortality and increased need for surgical intervention [57,102]. Determining a clinically relevant platelet threshold may help guide treatment decisions. Platelet indices, such as MPV, PMI, and PLCT, are important adjuncts to platelet count in the evaluation of neonates with NEC [194]. These parameters provide a more detailed picture of platelet morphology, activation, and turnover [195]. In conditions such as systemic inflammation and oxidative stress, the bone marrow attempts to compensate for platelet destruction by producing larger platelets, which is reflected in changes in MPV values [196] due to excessive inflammation [29].

Studies in neonates have shown that higher MPV values are associated with the development of NEC. For example, a study by Cekmez et al. [76] found that neonates who developed NEC had significantly higher MPV values both at birth and 72 h later. Zhang et al. [29] further identified that MPV and PDW are independent markers for predicting surgical NEC, with increased values being associated with disease severity and prognosis. Furthermore, Meng et al. [48] highlighted that the combination of MPV, PDW, and other markers such as PCT may help predict the severity of NEC in preterm neonates, aiding in early diagnosis and intervention.

Monitoring platelet indices, such as MPV, PDW, and PLCT, in neonates with NEC provides valuable diagnostic and prognostic information. Changes in these markers reflect alterations in platelet function, activation, and turnover, which are critical for understanding the pathophysiology of NEC [195]. Additionally, low platelet counts are associated with an increased risk of developing NEC, disease progression, adverse outcomes such as intestinal perforation, and mortality. While platelet transfusions can temporarily increase platelet counts and reduce bleeding risks, they are also associated with potential risks, such as infections and changes in immune function [197]. Therefore, the decision to administer platelet transfusions should be made cautiously, considering both the benefits and the risks.

Overall, platelet indices play a crucial role in the diagnosis and management of NEC in neonates. Monitoring these indices, along with platelet counts, can assist in early diagnosis, risk assessment, and treatment planning, helping clinicians optimize care and improve outcomes for affected neonates.

Neutrophils play a vital role in the body’s immune defense by responding to bacterial infections through various mechanisms, such as the release of antimicrobial substances and the enhancement of macrophage chemotaxis [12]. In neonates, neutropenia is often associated with adverse outcomes, such as NEC-related mortality [44]. Neutrophil-related parameters, such as ANC, also play an important role in the prognosis of NEC, with higher counts being associated with poor outcomes, as demonstrated by Gordon et al. [103]. Several studies have shown that a decrease in the ANC in the early stages of NEC is a predictor of adverse outcomes, including the need for surgical intervention [44,72]. An elevated NLR has also been associated with the severity of NEC, although no universally accepted cutoff values for NLR in the diagnosis of NEC have been established [45]

Lymphocytes play a key role in regulating the immune response and are recruited during the inflammatory response in neonates with NEC [12]. Lymphopenia is commonly observed in neonates with severe NEC and is associated with a poorer prognosis, particularly in those requiring surgical intervention [66,72]. A decrease in the ALC within 24–72 h of NEC onset is associated with progression to FNEC and increased mortality [82]. Early measurement of ALC is useful in predicting disease severity, particularly in preterm neonates with surgical NEC [67,103]. The platelet-to-lymphocyte ratio (PLR) has been proposed as a potential biomarker for NEC, with elevated values associated with more severe disease forms [30]. Recent studies suggest that combining the PLR and the NLR can improve the accuracy of sepsis diagnosis [178]. Similarly, further studies are needed to assess their accuracy in NEC prediction. However, further multicenter studies are necessary to validate these findings and integrate them into clinical practice.

Eosinophils are involved in inflammatory processes and tissue repair, but their role in the pathophysiology of NEC remains unclear [198]. Eosinophilia is observed in preterm neonates with NEC, often in association with transfusions, infections, and microbiota alterations [90]. Although eosinophilia is a common hematologic change in neonates with NEC, it does not appear to be associated with disease severity or the need for surgery [46,60]. However, persistent eosinophilia has been linked to adverse outcomes, such as intestinal strictures and liver fibrosis in some cases [199].

Monocytes play an important role in the immune response, and their depletion in patients with NEC is noteworthy [105]. Monocytopenia is a distinctive hematologic feature of NEC and may serve as an early marker for the disease [77]. Several studies have shown that a significant drop in AMC from baseline can distinguish the stages of NEC, as well as differentiate surgical from medical NEC [49,105]. A decrease in AMC is associated with adverse outcomes [49].

Additionally, anemia is a key factor in the prognosis of NEC, with lower hemoglobin and hematocrit levels being associated with severe NEC and increased risk of mortality [103]. Premature infants often develop anemia, primarily due to hematopoietic immaturity, erythropoietin deficiency, and rapid growth, which can compromise tissue oxygenation and increase oxidative stress, leading to adverse effects such as tissue damage and metabolic dysfunction [55].

There is also ongoing research into the role of RBC transfusions in the development of NEC. Some studies suggest that transfusions can worsen NEC, particularly in premature infants, although a causal relationship remains unclear [79]. The complexity of distinguishing between the effects of anemia and transfusions on NEC complicates the understanding of this association [117]. Studies suggest that pre-transfusion anemia may increase the risk of developing NEC, especially in very low birth weight (VLBW) infants. Song et al. [101] found that severe anemia within 72 h of birth was a significant risk factor for NEC in VLBW infants. Similarly, Singh et al. [43] showed that a decrease in hematocrit levels raised the risk of early-onset NEC. Patel et al. [46] observed a six-fold increase in NEC incidence in VLBW infants with hemoglobin levels < 8 g/dL. While RBC transfusions are common in these infants, research continues to investigate whether transfusions themselves or the pre-existing anemia contribute to NEC. These findings highlight the need for further studies, particularly large-scale randomized trials, to clarify the complex relationship between anemia, transfusions, and NEC development.

The absence of reliable diagnostic methods is one of the most important challenges in diagnosing NEC. In contrast to many other conditions in modern medicine, NEC diagnosis is largely clinical and relies on patient history, physical examination, and laboratory findings. The difficulty in detecting this high-mortality disease with greater accuracy results in persistently high fatality rates. Physical examination and abdominal radiographs remain essential diagnostic tools; however, their limitations are well recognized. Biochemical and hematological markers, such as thrombocytopenia and acidosis, provide valuable insights in advanced disease stages but lack sufficient specificity for early detection. Given that early and accurate diagnosis is important for improving outcomes, the need for more sensitive and specific diagnostic tools remains on ongoing concern in the management NEC.

Incorporating hematological parameters into predictive models for NEC has shown promising results. CBC parameters such as anemia, thrombocytopenia, and neutropenia can significantly improve prognostic accuracy, but most models fail to account for dynamic changes in these markers. More sophisticated models that incorporate longitudinal changes in hematological parameters are necessary. Premature infants are particularly vulnerable to NEC, which is associated with high mortality and long-term complications. Accurate predictive models are essential for early intervention and personalized treatment. Recent studies have developed models that integrate clinical, laboratory, and radiological data to predict NEC progression, the need for surgical intervention, and outcomes [37,62,65,81,94].

Additionally, the role of biomarkers such as PLR and NLR has garnered attention. Both PLR and NLR have been proposed as potential predictors of NEC severity and prognosis, with elevated values often associated with poor outcomes. Including these ratios in predictive models may enhance their accuracy by reflecting the balance between inflammation and immune response, which are critical factors in the pathophysiology of NEC. However, further studies are needed to validate their incorporation into standardized NEC prediction models.

## 11. Study Limitation

An important limitation of this review arises from the variability among the included studies. Ideally, a comprehensive evaluation of high-quality evidence would involve only randomized controlled trials (RCTs), considered the gold standard for assessing interventions. However, we were unable to identify any RCTs addressing the topic. As a result, we included both prospective and retrospective observational studies in our systematic review. Furthermore, the significant heterogeneity among the included studies—such as differences in populations (e.g., preterm, low birth weight, extremely low birth weight), measured CBC parameters, and study endpoints—represented a major limitation, deeming a meta-analysis inappropriate. Finally, due to our selection criteria, only studies published in English were included, which may introduce language bias and should be acknowledged. It is important to note that unpublished studies and preprints were not included in this review. As a result, the potential for publication bias exists, as studies with null or negative results are less likely to be published in peer-reviewed journals. While we acknowledge this limitation, the scope of our review and the resources available for screening unpublished data restricted our ability to include such sources. This exclusion may have impacted the comprehensiveness of our findings.

## 12. Conclusions

Despite important progress in recent years, NEC remains a complex and life-threatening condition, highlighting the crucial importance of timely recognition. Our literature review underlines a wider effort to identify reliable biomarkers within the CBC panel. CBC parameters have proven to be valuable potential tools for clinicians, aiding the diagnostic and prognostic insights that often precede NEC onset. Parameters such as thrombocytopenia, neutropenia, anemia, PLR, and NLR emerge as significant prognostic indicators. However, further prospective studies are needed to confirm their appropriate inclusion in routine NICU practice. Such validation could aid in the adoption of individualized treatment approaches, eventually improving outcomes for neonates affected by NEC.

The lack of reliable diagnostic methods remains a major challenge, as NEC diagnosis relies primarily on clinical assessment and imaging, both of which have limitations. Hematological markers, such as thrombocytopenia and acidosis, offer useful insights but lack specificity for early detection. Given the high mortality rates, there is an urgent need for more sensitive and specific diagnostic tools. Our review revealed an important gap and emerging need for more studies focused on developing a scoring system that combines clinical, laboratory, and radiologic data to predict NEC progression, the need for surgical intervention, and overall prognosis. However, further research is required to validate CBC markers in clinical models and develop more reliable prognostic tools. The incorporation of multifactorial approaches may improve NEC management and contribute to reducing mortality and disease-related complications.

## Figures and Tables

**Table 1:** Studies Focused on Hematologic Parameters Associated with NEC Mortality.

First Author	Year		Total Sample	NEC Sample	Outcome Measured	Complete Blood Count (CBC) Parameters Assessed	Comments-Results
Hutter et al. [26]	1976	Cohort study	40	40	To outline hematologic abnormalities and their possible clinical significance in neonates with NEC.	PLT count, granulocyte count and WBC count	Although the platelet count tended to be lower in the neonates who died, no statistically significant (*p* = 0.10) difference was noted between the groups, whereas the difference between the mean of absolute granulocyte count was found to be statistically significant (*p* = 0.01).
Dykes et al. [47]	1985	Retrospective	80	80	To identify objective prognostic factors in neonates with NEC.	WBC, neutrophils, lymphocytes, Hb, PLT, Hct	Stepwise logistic regression analysis identified pH value, platelet count, and the presence of congenital defects as independent predictors of outcomes in neonates with NEC.
McCormack et al. [62]	1987	Retrospective	54	54	To assess clinical, radiologic and laboratory data in predicting the severity of NEC from the initial presentation in neonates.	Hct, WBC, PLT, WBC immature forms	A scoring system was developed to predict the severity of NEC based on six factors: days preceding enteral feeding, blood pH, serum bicarbonate, WBC differential, abdominal tenderness and portal vein gas. A score of ≥3 indicates a higher risk of severe NEC, with a mortality rate exceeding 50%.
Ragazzi et al. [59]	2003	Retrospective	232	232	To assess whether the initial full blood count (after NEC diagnosis) is a useful predictor for NEC outcome and disease severity.	Neutrophils, platelets and their product (PN product)	The initial platelet counts and initial platelet–neutrophil (PN) product were significantly lower in non-survivors compared to survivors. The ROC curve analysis showed that the PN product did not outperform the platelet count alone in predicting mortality. However, ROC analysis demonstrated that the PN product (AUC: 0.69) was a better predictor of disease extent in NEC patients than either platelet count alone (AUC: 0.65) or neutrophil count alone (AUC: 0.64).
Kenton et al. [100]	2005	Retrospective study	91	91	To study whether the severity and timing of severe thrombocytopenia (platelet count <100,000/mm^3^) can serve as a predictor for adverse outcomes in infants with NEC.	PLT count	The onset of severe thrombocytopenia within the first 3 days following the diagnosis of NEC is associated with an increased risk of bowel gangrene, higher morbidity, and mortality.
Kessler et al. [37]	2006	Retrospective	128	128	Surgical intervention, NEC severity and survival based on early laboratory parameters	hemoglobin, leukocyte and platelet counts,	A NEC score is proposed, incorporating Lactate levels, GA, Bell’s stage and PLT counts (even though alone there was no significant difference between study groups, it increased the predictive power of the score).
Al Tawil et al. [99]	2013	Retrospective case control	150	32	NEC development prediction according to CBC results	PLT counts	The presence of thrombocytopenia was associated with a poor prognosis and increased risk of mortality (OR = 33.6, 95%: 3.43–328.9)
Atici et al. [63]	2014	prospective cohort study	31	31	NEC prognosis based on clinical and laboratory parameters	hemoglobin, hematocrit, white blood cell, neutrophil, lymphocyte and platelet counts	Thrombocytopenia was the only CBC parameter related to mortality. Optimal cut-off level was 110,000/µL (with 93.3% sensitivity and 87.5% specificity AUC = 0.838, 95% CI: 0.667–1.008; *p* = 0.001).
Gordon et al. [103]	2016	Retrospective cohort study	5166	5166	NEC mortality predicted by CBC parameters on the day of diagnosis	Hct-Hb-PLT-WBC-segmented neutrophils-and bands, lymphocytes-Eosinophils (percentages and absolute counts)	In neonates who died the total WBC, absolute neutrophil count and segmented neutrophils and bands (Segs/bands) were higher; but the hemoglobin/hematocrit, absolute monocyte count, absolute eosinophil count and platelet count were lower, compared to those who lived. In a multivariate analysis: decreased PLT counts, higher absolute MON-LYMPH count, Segs/bands > 0.2 were identified as the most important hematologic factors associated with death. Low platelet counts (≤150,000) and severe anemia at NEC onset were associated with a higher risk of mortality across all gestational ages.
Kordazs et al. [97]	2021	Retrospective multicenter study	157	157	Difference in CBC parameters between survivors-non survivors, severe—non severe NEC	CBC parameters assessed	Low Hgb levels correlated with severe NEC and mortality. A proportion of immature neutrophils above 34% at disease onset was found to be associated with NEC stage III (OR 2.9, 1.2–7.4, *p* = 0.025), while a WBC count higher than 22 g/L during the course of disease was correlated with severe NEC (OR 4, 1.8–9.3, *p* < 0.001).
Siahaan et al. [84]	2021	Retrospective study	52	52	To assess survival of neonates with NEC and associate it with the prognostic factors	PLT	Platelet count was not significantly associated with the survival of neonates with NEC.
Feng et al. [55]	2022	Retrospective study	114	114	To evaluate the potential of PLT count to predict NEC surgery and mortality.	PLT, WBC, Hb	Surgical NEC was significantly associated with decreased WBC counts (median: 8.93 versus 10.19 ×10^9^/L, *p* = 0.041), HB levels (mean: 133.20 versus 146.03 g/L, *p* = 0.035), and PLT counts (median: 163.0 versus 328.0 ×10^9^/L, *p* < 0.001). PLT counts were identified as an independent predictor for the need for surgery in NEC patients (OR = 0.995, 95% CI: 0.990~0.999, *p* = 0.029; AUC: 0.763).
Qin et al. [44]	2022	Retrospective cohort study	157	157	Risk factors of severe surgical NEC and mortality.	absolute neutrophil count (ANC), before and at the onset of NEC, difference in absolute neutrophil count (ΔANC) at NEC onset, and platelet counts	A decrease in neutrophil count was the most sensitive predictive factor for severe surgical NEC and death, especially when combined with PLT counts. Even when adjusted for multiple confounders for each 10^9^/L ΔANC reduction at NEC onset, the odds for severe NEC increased by almost 25% (OR 1.308, 95% CI 1.113–1.539; *p* = 0.001).
Elmoneim et al. [92]	2022	Retrospective	188	NR	To assess the risk factors for the development of NEC and other co morbidities.	PLT counts, PLT counts drop > 30% within 7 days	The odds of having NEC were significantly higher (*p* < 0.01) in preterm neonates who had ≥ 30% decrease in platelet counts either with or without thrombocytopenia than those of thrombocytopenic preterm neonates with no decline in platelets decline.
Han et al. [39]	2022	Retrospective	271	271	To examine the surgical outcomes in neonates with perforated versus non-perforated NEC and determine the criteria for surgical intervention.	PLT, WBC	CBC parameters were similar among 2 groups (perforated and non-perforated group). Infants with surgical NEC in the non-perforated group were more prone to bowel necrosis, and their mortality rate was higher than that in the perforated group.
Zouari et al. [89]	2023	Retrospective study	102	102	To evaluate the predictive factors for mortality in patients with NEC.	CBC, PLT	Gestational age < 32 weeks, Apgar score < 8 at 5 min, very low birth weight, severe thrombocytopenia, Bell’s stage 3 and sepsis during hospitalization were identified as predictive factors for mortality in neonates with NEC.
Assenga and Tooke [79]	2024	retrospective observational cohort	1582	104	To assess the proportion, patterns, and risk factors associated with mortality in VLBW neonates diagnosed with NEC in a middle-income setting.	complete blood count	Anaemia necessitating blood transfusion (*p* = 0.003) and thrombocytopenia requiring platelet transfusion (*p* = 0.033) were identified as significant factors linked to increased mortality in NEC cases.

**Table 2:** Studies exploring the association between anemia and NEC development and severity.

First Author	Year		Total Sample	NEC Sample	Outcome Measured	Complete Blood Count (CBC) Parameters Assessed	Comments-Results
Dykes et al. [47]	1985	Retrospective	80	80	To identify objective prognostic factors in neonates with NEC.	WBC, neutrophils, lymphocytes, hemoglobin (Hb), PLT, hematocrit (Hct)	Stepwise logistic regression analysis identified pH value, platelet count, and the presence of congenital defects as independent predictors of outcomes in neonates with NEC.
McCormack et al. [53]	1987	Retrospective	54	54	To assess clinical, radiologic and laboratory data in predicting the severity of NEC from the initial presentation in neonates.	Hct, WBC, PLT, WBC immature forms	A scoring system was developed to predict the severity of NEC based on six factors: days preceding enteral feeding, blood pH, serum bicarbonate, WBC differential, abdominal tenderness and portal vein gas. A score of ≥3 indicates a higher risk of severe NEC, with a mortality rate exceeding 50%.
Mandel et al. [75]	2004	case control	46	23	To evaluate whether nucleated red blood cell (NRBC), counts and other CBC parameters can be associated with the development of NEC.	Hct, WBC, PLT, NRBC at birth	NRBC was the only CBC parameter that was associated with NEC.
Kessler et al. [37]	2006	Retrospective	128	128	Surgical intervention, NEC severity and survival based on early laboratory parameters	hemoglobin, leukocyte and platelet counts,	A NEC score is proposed, incorporating Lactate levels, GA, Bell’s stage and PLT counts (even though alone there was no significant difference between study groups, it increased the predictive power of the score).
Hallstom et al. [66]	2006	Prospective study	78	26	Laboratory differences predicting NEC development in preterm neonates < 33 gestational age (GA)weeks	hemoglobin, hematocrit, platelet and leukocyte counts; I/T ratios (immature neutrophil count as a proportion of the total neutrophil count)	Based on the analysis of variance for repeated measures, the hemoglobin concentration (*p* = 0.006), hematocrit levels (*p* = 0.038) and platelet counts (*p* = 0.029) were significantly lower, while leukocyte counts (*p* = 0.016) and I/T ratio (*p* = 0.014) were significantly higher in neonates with NEC grade II to III in comparison to the control group.
Lambert et al. [66]	2011	Case -control	271,327	523	To determine the risk factors associated with fulminant NEC.	WBC, I/T, Hb, Hct, lymphocyte	Portal venous gas, anemia, rapid escalation of feeding, an elevated I/T neutrophil ratio (>0.5), a low lymphocyte count (<4000/μL), and recent increases in fortifier intake may all be linked to the development of fulminant NEC.
Singh et al. [43]	2011	case control	333	111	To determine the association between anemia, RBC transfusions, and the development of NEC in preterm infants.	Hct	Anemia is linked to a higher likelihood of developing NEC in preterm infants, with the risk escalating as anemia becomes more severe. Additionally, RBC transfusions may contribute to a greater likelihood of NEC, and this connection seems to follow a temporal pattern. This relationship remains significant even when adjusting for “transfusion propensity” in a multivariable model that accounts for hematocrit (Hct) levels and other crucial clinical variables.
Cekmez et al. [76]	2013	Prospective case control	272	21	Difference in MPV among NEC (and other infant diseases)-controls	MPV-platelet count	MPV was significantly higher in infants with NEC (8.6 ± 0.7 fl) in comparison to controls when measured on the 1st day of life. High MPV value in the first hours of life was identified as a risk factor for the development of NEC, BPD and IVH in extremely preterm neonates.
Miner et al. [64]	2013	Retrospective multicenter study	220	220	Association of NEC severity with laboratory parameters	Hct, WBC, neutrophiI, lymphocyte, PLT, I/T, MPV	Preterm infants with earlier gestational age, lower birth weight, previous RBC transfusions, absence of early colostrum feedings, acidosis, abnormal CBC, elevated CRP, and sepsis are at higher risk of developing severe NEC requiring surgery.
Atici et al. [63]	2014	prospective cohort study	31	31	NEC prognosis based on clinical and laboratory parameters	hemoglobin, hematocrit, white blood cell, neutrophil, lymphocyte and platelet counts	Thrombocytopenia was the only CBC parameter related to mortality. Optimal cut-off level was 110,000/µL (with 93.3% sensitivity and 87.5% specificity AUC = 0.838, 95% CI: 0.667–1.008; *p* = 0.001).
Sho et al. [33]	2014	Retrospective case control	157	157	Ability of laboratory and clinical parameters to predict NEC totalis	Complete blood count (WBC, RBC, hemoglobin, hematocrit, MCV, MCH, MCHC, RDW, MPV, platelets), differential and absolute blood counts (neutrophils, bands, lymphs, monocytes, basophils, eosinophils, atypical lymphs, metamyelocytes, myelocytes and nucleated RBCs)	The presence of thrombocytopenia was the strongest independent risk factor and was associated with over an 84-fold higher risk of NEC-totalis OR = 84.3, 95%: [2.67, 2670].
Banerjee et al. [70]	2015	Retrospective	890	195	To investigate if the hemoglobin level at birth is linked to short-term outcomes in preterm infants born at or before 32 weeks of gestation.	Hb	Hb at birth was not a statistically significant factor for NEC when adjusted for BW and GA. Increased Hb level at birth by delaying umbilical cord clamping has been demonstrated to reduce the risk of NEC
Patel et al. [46]	2016	prospective, multicenter observational cohort study	598	44	To investigate the association between RBC transfusion, severe anemia, and the occurrence of NEC.	Hemoglobin levels	VLBW neonates with severe anemia (hemoglobin level lower than 8 g/dL) at a given week, had a higher estimated rate of NEC compared to those without severe anemia (adjusted cause-specific HR, 5.99 [95% CI, 2.00–18.0]; *p* = 0.001). RBC transfusion did not correlate with the overall incidence of NEC.
Gordon et al. [103]	2016	Retrospective cohort study	5166	5166	NEC mortality predicted by CBC parameters on the day of diagnosis	Hct-Hb-PLT-WBC-segmented neutrophils-and bands, lymphocytes-Eosinophils (percentages and absolute counts)	In neonates who died the total WBC, absolute neutrophil count and segmented neutrophils and bands (Segs/bands) were higher; but the hemoglobin/hematocrit, absolute monocyte count, absolute eosinophil count and platelet count were lower, compared to those who lived. In a multivariate analysis: decreased PLT counts, higher absolute MON-LYMPH count, Segs/bands > 0.2 were identified as the most important hematologic factors associated with death. Low platelet counts (≤150,000) and severe anemia at NEC onset were associated with a higher risk of mortality across all gestational ages.
Zhang et al. [88]	2017	Retrospective multicenter study	188	186	Stricture development in association with multiple baseline clinical and laboratory data	WBC, PLT, Hb, plateletcrit at disease onset	Neonates with stricture exhibited significantly elevated levels of C-reactive protein (CRP), white blood cells (WBC), and plateletcrit, with these increased levels persisting until the stricture resolved.
Lin et al. [51]	2019	Retrospective	352	352	To identify clinical and laboratory factors risk factors related to neonatal fulminant NEC and to develop a scoring system to identify patients at risk for NEC-totalis at the time of presentation.	Hct, PLT, WBC, neutrophil counts	To identify clinical and laboratory factors that differentiate NEC-totalis from other types of NEC and to create a scoring system to identify patients at risk for NEC-totalis at the time of presentation.
Cai et al. [36]	2020	Retrospective study	80	11	NEC development prediction according to CBC results.	PLT, WBC, Hb	The decrease in hemoglobin concentration, and rates of red blood cell transfusion and ventilator application were significantly higher in the NEC group than in the non-NEC group (all *p* < 0.05), while no significant differences in the WBC count, platelet count, and hemoglobin concentration, and blood culture were noted among the two groups.
Haefeli et al. [98]	2020	Retro case control	78	26	To identify risk factors for NEC in neonates with a significant patent ductus arteriosus (PDA)	Hb, WBC, PLT before NEC onset	NEC patients had lower Apgar scores (1′), higher incidence of congenital malformations, higher minimum platelet counts, and elevated CRP values prior to NEC onset, with higher mortality rates (29% vs. 2%, *p* < 0.001),
Kordazs et al. [97]	2021	Retrospective multicenter study	157	157	Difference in CBC parameters between survivors-non survivors, severe—non severe NEC	CBC parameters assessed	Low Hgb levels correlated with severe NEC and mortality. A proportion of immature neutrophils above 34% at disease onset was found to be associated with NEC stage III (OR 2.9, 1.2–7.4, *p=* 0.025), while a WBC count higher than 22 G/L during the course of disease was correlated with severe NEC (OR 4, 1.8–9.3, *p* < 0.001).
Song J et al. [65]	2021	Retrospective study	447	296	A novel metaheuristic algorithm was proposed to predict NEC diagnosis and prognosis.	white blood cell count, lymphocyte percentage, and mean platelet volume, Hb.	A feature selection and classification algorithm using pre-disease data for diagnostic classification and NEC risk prediction was developed. Neutrophil percentage, breast milk, probiotics, MCH, and anemia-RBC transfusion were identified as key predictors for classic and surgical NEC, playing a significant role in early diagnosis and risk assessment.
Feng et al. [40]	2022	Retrospective study	114	114	To evaluate the potential of PLT count to predict NEC surgery and mortality.	PLT, WBC, Hb	Surgical NEC was significantly associated with decreased WBC counts (median: 8.93 versus 10.19×10^9^/L, *p* = 0.041), HB levels (mean: 133.20 versus 146.03 g/L, *p* = 0.035), and PLT counts (median: 163.0 versus 328.0×10^9^/L, *p* < 0.001). PLT counts were identified as an independent predictor for the need for surgery in NEC patients (OR = 0.995, 95% CI: 0.990~0.999, *p* = 0.029; AUC: 0.763).
Song et al. [101]	2022	case control	467	467	To investigate the relationship between severe anemia, red blood cell transfusions, and the development of NEC in neonates.	Hemoglobin	In very low birth weight (VLBW) neonates, after adjusting for other variables, severe anemia within 72 h (OR = 2.404, *p* = 0.016), RBC transfusion within 24 h (OR = 4.905, *p* = 0.016), within 48 h (OR = 5.587, *p* = 0.008), and within 72 h (OR = 2.858, *p* = 0.011) were associated with an increased risk of developing NEC.
Garg et al. [72]	2022		336	336	To assess whether hematological profiles and transfusion patterns following the onset of NEC can help identify infants at risk of developing severe, fatal NEC.	CBC, hematocrit, hemoglobin, PLT	Neonates with fulminant NEC often exhibited thrombocytopenia, lymphopenia, neutropenia, and leukopenia. Additionally, those who received red blood cell transfusions after NEC onset or platelet transfusions before its onset were more likely to develop the fulminant form of the disease.
Chen et al. [90]	2023	Retrospective	216	216	To develop a prediction model of the rapid progression (Rp) of NEC in preterm neonates.	white blood cell count, hemoglobin, neutrophil count	White blood cell count < 5 ×10^9^/L, hemoglobin < 100 g/L, neutrophil count < 2 × 10^9^/L, pH < 7.3, and abnormal coagulation were positively correlated with RP-NEC in the invariable regression analysis, while in multivariable regression NEUT < 2.000 was the only CBC parameter difference between the two groups (most common in RpNEC).
Yu et al. [87]	2023	Retrospective	267	267	To explore the high-risk factors for surgical NEC.	erythrocytes, hemoglobin, white blood cells, platelets	Lower leukocytes (*p* = 0.001), lymphocytes (*p* < 0.001), erythrocytes (*p* = 0.004), and platelets counts (*p* = 0.039) were noted in neonates with surgical NEC. The multivariate logistic regression analysis identified lymphocytes counts as a potentially protective factor for surgical NEC (OR = 0.749; 95% CI: 0.588–0.954; *p* = 0.019).
Li et al. [82]	2023	Retrospective	206	206	To develop and assess a predictive nomogram for FNEC.	Hb, WBC, neutrophil, lymphocyte, eosinophils and monocyte counts at 3 intervals: 24 h before, at onset, 24 h after NEC	Neutrophil counts on the day of NEC onset as well as neutrophil, lymphocyte, and monocyte counts on day 1 after NEC onset along with other parameters (assisted ventilation after NEC onset, shock at NEC onset) were identified as the most relevant variables and were included in a predictive model for FNEC, which exhibited good discrimination capacity (AUC: 0.884; 95% CI 0.825–0.943).
Jiang et al. [42]	2023	Retrospective cohort	155	155	To assess if the sepsis, anemia, and PLT activation index are vital NEC predictors in LBW neonates.	Hb levels, PLT indicators (such as PLT count, PDW, MPV, plateletcrit, and PLCR)	In LBW neonates without sepsis, anemia [*p* = 0.001, odds ratio (OR) = 4.367, 95% confidence interval (CI): 1.853–10.291], high PLCR values (*p* < 0.001, OR = 2.222, 95% CI: 1.633–3.023), and high PCT values (*p* =0.024, OR = 1.368, 95% CI: 1.042–1.795) increased the risk of NEC; AUC of PLCR, sensitivity, specificity, and cutoff value were 0.739, 0.770, 0.610, and 33.55, respectively.
Dantes et al. [53]	2024	Retrospective cohort study	338	69	To examine the clinical characteristics associated with SIP and NEC diagnosis and develop a scoring algorithm for accurate preoperative diagnosis.	CBC parameters	The differences in CBC parameters were not statistically significant in aiding the differentiation between SIP and surgical NEC. A risk score was developed using statistically significant parameters (pneumatosis, abdominal wall erythema, higher ALD and history of feeds)
Chong et al. [68]	2024	Retrospective study	229	229	To identify age-specific hematological biomarkers that could predict surgical NEC.	WBC, hemoglobin; Hct, PLT, ANC, absolute lymphocyte count; absolute monocyte count; absolute eosinophil count; ABC: absolute basophil count	Patients with NEC show distinct hematological characteristics based on GA, and independent predictors of surgical NEC vary across different GAs.
Li et al. [93]	2024	Retrospective study	334	334	To develop and establish a predictive model for RP-NEC.	CBC parameters	Plasma sodium levels < 135 mmol/L, C-reactive protein ≥ 10 mg/L, platelet count < 100 × 10^9^/L, lymphocyte count < 1.5 × 10^9^/L, blood pH < 7.2, and the presence of ascites at the onset of NEC were identified as independent risk factors for RP-NEC and were incorporated in the predictive model, which demonstrated an AUC value of 0.983 (95% CI 0.97–0.99).

Abbreviations: Absolute Lymphocyte Count, ALC; Absolute Eosinophil Count, AEC; Absolute Monocyte Count, AMC; Absolute Neutrophil Count, ANC; Area Under the Curve, AUC; Complete Blood Count, CBC; C-Reactive Protein, CRP; Fulminant Necrotizing Enterocolitis, FNEC; Gestational Age, GA; Hematocrit, Hct; Hemoglobin, Hb; Immature/Total Neutrophil Ratio, I/T; Low Birth Weight, LBW; Mean Corpuscular Hemoglobin, MCH; Mean Corpuscular Hemoglobin Concentration, MCHC; Mean Corpuscular Volume, MCV; Mean Platelet Volume, MPV; Necrotizing Enterocolitis, NEC; Nucleated Red Blood Cell, NRBC; Odds Ratio, OR; Patent Ductus Arteriosus, PDA; Rapidly Progressive Necrotizing Enterocolitis, RpNEC; Red Blood Cell, RBC; Red Cell Distribution Width, RDW; Very Low Birth Weight, VLBW; White Blood Cell, WBC.

**Table 3:** Studies Developing Predictive Models for Assessing NEC Severity and Prognosis in Neonates Using CBC Parameters.

First Author	Year		Total Sample	NEC Sample	Outcome Measured	Complete Blood Count (CBC) Parameters Assessed	Comments-Results
McCormack et al. [62]	1987	Retrospective	54	54	To assess clinical, radiologic and laboratory data in predicting the severity of NEC from the initial presentation in neonates.	Hct, WBC, PLT, WBC immature forms	A scoring system was developed to predict the severity of NEC based on six factors: days preceding enteral feeding, blood pH, serum bicarbonate, WBC differential, abdominal tenderness and portal vein gas. A score of ≥3 indicates a higher risk of severe NEC, with a mortality rate exceeding 50%.
Gupta et al. [81]	1994	Retrospective	49	49	Surgical Intervention based on laboratory parameters measured at time intervals 0–4, 4–12, 12–24 and 24–36 h after diagnosis	WBC, immature: total neutrophil ratio (I:T), PLT count	A scoring scale with a good predictive value was developed in identifying patients who might require surgery, assigning one point for each of the following parameters: WBC < 9000/mm^3^, I:T > 0.5, PLT < 200,000/mm^3^, and BEs −2.
Kessler et al. [37]	2006	Retrospective	128	128	Surgical intervention, NEC severity and survival based on early laboratory parameters	hemoglobin, leukocyte and platelet counts,	A NEC score is proposed, incorporating Lactate levels, GA, Bell’s stage and PLT counts (even though alone there was no significant difference between study groups, it increased the predictive power of the score).
Lin et al. [51]	2019	Retrospective	352	352	To identify clinical and laboratory factors risk factors related to neonatal fulminant NEC and to develop a scoring system to identify patients at risk for NEC-totalis at the time of presentation.	Hct, PLT, WBC, neutrophil counts	To identify clinical and laboratory factors that differentiate NEC-totalis from other types of NEC and to create a scoring system to identify patients at risk for NEC-totalis at the time of presentation.
Song et al. [45]	2021	Retrospective study	447	296	A novel metaheuristic algorithm was proposed to predict NEC diagnosis and prognosis.	white blood cell count, lymphocyte percentage, and mean platelet volume, Hb.	A feature selection and classification algorithm using pre-disease data for diagnostic classification and NEC risk prediction was developed. Neutrophil percentage, breast milk, probiotics, MCH, and anemia-RBC transfusion were identified as key predictors for classic and surgical NEC, playing a significant role in early diagnosis and risk assessment.
Feng et al. [55]	2022	Retrospective study	131	131	To evaluate the association of SII with surgical risk in neonates with NEC.	WBC, neutrophil count, NLR, PLR, SII	A prediction model based on the combination of Low-SII and Low-PLR resulted in an AUC of 0.838 (95% CI: 0.764–0.897, *p* < 0.001) indicating a good predictive performance for identifying the patients who received surgical intervention.
Li et al. [95]	2022	Retrospective stydy	207	207	To identify predictors for bowel resection in neonates diagnosed with NEC.	CBC parameters assessed	An increased incidence of neutropenia [*p* = 0.004]) at disease diagnosis in neonates with NEC was associated with bowel loss, suggesting a severe case of NEC. Neutrophil, counts on day 1 after NEC onset along with other parameters (birth weight < 2520 g, hypotension, pneumoperitoneum, acidosis, and intestinal wall thickness > 1.08 mm) were identified as most key variables and were included in a predictive model for severe NEC.
Chen et al. [90]	2023	Retrospectve	216	216	To develop a prediction model of the rapid progression (Rp) of NEC in preterm neonates.	white blood cell count, hemoglobin, neutrophil count	White blood cell count < 5 × 10^9^/L, hemoglobin < 100 g/L, neutrophil count < 2 × 10^9^/L, pH < 7.3, and abnormal coagulation were positively correlated with RP-NEC in the invariable regression analysis, while in multivariable regression NEUT < 2.000 was the only CBC parameter difference between the two groups (most common in RpNEC).
Li et al. [82]	2023	Retrospective	206	206	To develop and assess a predictive nomogram for FNEC.	Hb, WBC, neutrophil, lymphocyte, eosinophils and monocyte counts at 3 intervals: 24 h before, at onset, 24 h after NEC	Neutrophil counts on the day of NEC onset as well as neutrophil, lymphocyte, and monocyte counts on day 1 after NEC onset along with other parameters (assisted ventilation after NEC onset, shock at NEC onset) were identified as the most relevant variables and were included in a predictive model for FNEC, which exhibited good discrimination capacity (AUC: 0.884; 95% CI 0.825–0.943).
Dantes et al. [53]	2024	Retrospective cohort study	338	69	To examine the clinical characteristics associated with SIP and NEC diagnosis and develop a scoring algorithm for accurate preoperative diagnosis.	CBC parameters	The differences in CBC parameters were not statistically significant in aiding the differentiation between SIP and surgical NEC. A risk score was developed using statistically significant parameters (pneumatosis, abdominal wall erythema, higher ALD and history of feeds)
Guo et al. [41]	2024	Retrospective study	191	191	To assess the predictive value of CBC parameters, CRP, and PCT in determining the severity of NEC, and to develop a model for distinguishing surgically treated NEC from non-surgically treated NEC.	PLR and the combination of WBC, ANC, ALC, NLR	Elevated PLR is linked to severe inflammation in patients with surgical or fatal NEC. The predictive model combining ANC, PLR, CRP, and PCT can distinguish surgical/fatal NEC from medical NEC.
Huang et al. [94]	2024	Retrospective study	160	160	To investigate the clinical characteristics of NEC complicated by intestinal perforation, identify associated risk factors, develop effective early predictors, and construct a visual scoring system for independent risk variables to provide a scientific basis for reducing morbidity and mortality in neonates with NEC.	PLT count	Thrombocytopenia and hypoalbuminemia may serve as independent risk factors for predicting intestinal perforation in neonates with NEC.
Li et al. [93]	2024	Retrospective study	334	334	To develop and establish a predictive model for RP-NEC.	CBC parameters	Plasma sodium levels < 135 mmol/L, C-reactive protein ≥ 10 mg/L, platelet count < 100 × 10^9^/L, lymphocyte count < 1.5 × 10^9^/L, blood pH < 7.2, and the presence of ascites at the onset of NEC were identified as independent risk factors for RP-NEC and were incorporated in the predictive model, which demonstrated an AUC value of 0.983 (95% CI 0.97–0.99).

Abbreviations: Absolute Lymphocyte Count, ALC; Absolute Neutrophil Count, ANC; Area Under the Curve, AUC; Complete Blood Count, CBC; Confidence Interval, CI; C-Reactive Protein, CRP; Fulminant Necrotizing Enterocolitis, FNEC; Gestational Age, GA; Hematocrit, Hct; Hemoglobin, Hb; Intraventricular Hemorrhage, IVH; Necrotizing Enterocolitis, NEC; Neutrophil/Lymphocyte Ratio, NLR; Platelet-to-Lymphocyte Ratio, PLR; Platelets, PLT; Rapidly Progressive Necrotizing Enterocolitis, RpNEC; Red Blood Cell, RBC; Spontaneous Intestinal Perforation, SIP; Systemic Immune-Inflammation Index, SII; White Blood Cell, WBC.

## Data Availability

Data are contained within the article.

## Supplementary Materials

The following supporting information can be downloaded at: https://www.mdpi.com/article/10.3390/jcm14072530/s1, Table S1: Characteristics of the Studies Included in the Systematic Review; PRISMA checklist.
