# Supplementary material for: Diagnostic and Prognostic Value of Hematological Parameters in Necrotizing Enterocolitis: A Systematic Review
Source: J Clin Med. 2025 Apr 7;14(7):2530. doi: 10.3390/jcm14072530 (PMC11989880; doi:10.3390/jcm14072530)
Supplement: Supplementary file 1 [file jcm-14-02530-s001.zip › jcm-3545929-supplementary.pdf]

Table S1. Characteristics of the Studies Included in the Systematic Review.

First author	Year	Country		Total Sample	NEC sample	Outcome measured	Complete blood count (CBC) parameters assessed	Comments-Results
Hutter et al [26]	1976	USA	Cohort study	40	40	To outline hematologic abnormalities and their possible clinical significance in neonates with necrotizing enterocolitis (NEC).	Platelet (PLT) count, granulocyte count and White Blood Cell count (WBC) count	Although the platelet count tended to be lower in the neonates who died, no statistically significant (P = 0.10) difference was noted between the groups, whereas the difference between the mean of absolute granulocyte count was found to be statistically significant (P = 0.01).
Dykes et al[47]	1985	Scotland	Retrospective	80	80	To identify objective prognostic factors in neonates with NEC.	WBC, neutrophils, lymphocytes, hemoglobin (Hb), PLT, hematocrit (Hct)	Stepwise logistic regression analysis identified pH value, platelet count, and the presence of congenital defects as independent predictors of outcomes in neonates with NEC.
McCormack et al[62]	1987	USA	Retrospective	54	54	To assess clinical, radiologic and laboratory data in predicting the severity of NEC from the initial presentation in neonates.	Hct, WBC, PLT, WBC immature forms	A scoring system was developed to predict the severity of NEC based on six factors: days preceding enteral feeding, blood pH, serum bicarbonate, WBC differential, abdominal tenderness and portal vein gas. A score of ≥3 indicates a higher risk of severe NEC, with a mortality rate exceeding 50%.
Gupta et al [81]	1994	USA	Retrospective	49	49	Surgical Intervention based on laboratory parameters measured at time intervals 0-4, 4-12, 12-24 and 24-36 hours after diagnosis	WBC, immature: total neutrophil ratio (I:T), PLT count	A scoring scale with a good predictive value was developed in identifying patients who might require surgery, assigning one point for each of the following parameters: WBC <9,000/mm3, I:T >.5, PLT <200,000/mm^3, and BEs -2.
Schober et al[96]	1994	Austria	Retrospective study	61	61	To identify risk factors or indices associated with the severity or progression of NEC, and to provide clearer guidelines for determining the need for surgical intervention.	WBC, PLT, neutrophils, lymphocytes, i/T ratio	Infants who underwent surgery had significantly higher counts of immature granulocytes and an elevated i:T ratio, along with lower counts of total granulocytes, lymphocytes, platelets, and smaller placental weights. However, when adjusted for gestational age, these parameters lost their significance in the group with a gestational age under 34 weeks and were more relevant in the group with a gestational age over 35 weeks.
Ververidis et al [102]	2001	UK	Retrospective study	58	58	Association of PLT count and PLT count drop with NEC severity	PLT counts	A platelet count less than 100×10^9/L is commonly observed in infants with NEC. A rapid decline in platelet count represents poor prognostic factors, indicating a more severe disease category.
Ragazzi et al [59]	2003	UK	Retrospective	232	232	To asses whether the initial full blood count (after NEC diagnosis) is a useful predictor for NEC outcome and disease severity.	Neutrophils, platelets and their product (PN product)	The initial platelet count and initial platelet–neutrophil (PN) product were significantly lower in non-survivors compared to survivors. The ROC curve analysis showed that the PN product did not outperform the platelet count alone in predicting mortality. However, ROC analysis demonstrated that the PN product (AUC: 0.69) was a better predictor of disease extent in NEC patients than either platelet count alone (AUC: 0.65) or neutrophil count alone (AUC: 0.64).
Mandel et al [83]	2004	Isreal	case control	46	23	To evaluate whether nucleated red blood cell (NRBC), counts and other CBC parameters can be associated with the development of NEC.	Hct, WBC, PLT, NRBC at birth	NRBC was the only CBC parameter that was associated with NEC.
Kenton et al [100]	2005	USA	Retrospective study	91	91	To study whether the severity and timing of severe thrombocytopenia (platelet count <100,000/mm^3) can serve as a predictor for adverse outcomes in infants with NEC.	PLT count	The onset of severe thrombocytopenia within the first 3 days following the diagnosis of NEC is associated with an increased risk of bowel gangrene, higher morbidity, and mortality.
Kessler et al [37]	2006	UK	Retrospective	128	128	Surgical intervention, NEC severity and survival based on early laboratory parameters	hemoglobin, leukocyte and platelet counts,	A NEC score is proposed, incorporating Lactate levels, GA, Bell's stage and PLT counts (even though alone there was no significant difference between study groups, it increased the predictive power of the score).
Hallstom et al [66]	2006	Finland	Prospective study	78	26	Laboratory differences predicting NEC development in preterm neonates <33 gestational age (GA)weeks	hemoglobin, hematocrit, platelet and leukocyte counts; I/T ratios (immature neutrophil count as a proportion of the total neutrophil count)	Based on the analysis of variance for repeated measures, the hemoglobin concentration (P = .006), hematocrit levels (P = .038) and platelet counts (P = .029) were significantly lower, while leukocyte counts (P = .016) and I/T ratio (P = .014) were significantly higher in neonates with NEC grade II to III in comparison to the control group.
Morag et al[[75]	2008	Canada	Retrospective case control study	190	23	To investigate the clinical factors and outcomes linked to leukocytosis in very low birth weight (VLBW) neonates.	WBC	Late-onset leukocytosis (WBC count of X40 000/ml) was associated with necrotizing enterocolitis, suggesting that the timing of leukocytosis in association with postnatal age should be taken into account when making clinical decisions.
Srinivasjois et al [32]	2010	Australia	Retrospective	37	37	Progression to surgery in confirmed NEC according to laboratory parameters	PLT	No significant difference in platelet count was noted between the neonates with medical NEC and those with surgical NEC (p ¼ 0.133)
Rastogi et al [57]	2011	USA	Retrospective study	286	NR	Association of decrease in PLT counts with clinical outcome of preterm neonates	PLT-PLT drop < or >30% between day 7 and day 28 of life	A ≥30% drop in platelet counts in both thrombocytopenic and non thrombocytopenic groups of preterm neonates was associated with increased risk for NEC developed.
Rastogi et al [78]	2011	USA	Retrispective cohort	286	NR	To assess the ability of thrombocytopenia severity, as graded by the National Cancer Institute (NCI) Common Toxicity Criteria, to predict the clinical outcomes of preterm neonates admitted to the NICU.	PLT	NCI thrombocytopenia Grade 2-3-4 were significantly associated with NEC, with the OR increasing in parallel with the severity of thrombopenia.
Lambert et al [66]	2011	USA	Case -control	271327	523	To determine the risk factors associated with fulminant NEC.	WBC, I/T, Hb, Hct, lymphocyte	Portal venous gas, anemia, rapid escalation of feeding, an elevated I/T neutrophil ratio (>0.5), a low lymphocyte count (<4000/μl), and recent increases in fortifier intake may all be linked to the development of fulminant NEC.
Singh et al [43]	2011	USA	case control	333	111	To determine the association between anemia, RBC transfusions, and the development of NEC in preterm infants.	Hct	Anemia is linked to a higher likelihood of developing NEC in preterm infants, with the risk escalating as anemia becomes more severe. Additionally, RBC transfusions may contribute to a greater likelihood of NEC, and this connection seems to follow a temporal pattern. This relationship remains significant even when adjusting for "transfusion propensity" in a multivariable model that accounts for hematocrit (Hct) levels and other crucial clinical variables.
Cekmez et al [76]	2013	Turkey	Prospective case control	272	21	Difference in MPV among NEC (and other infant diseases)- controls	MPV-platelet count	MPV was significantly higher in infants with NEC (8.6±0.7 fl) in comparison to controls when measured on the 1st day of life. High MPV value in the first hours of life was identified as a risk factor for the development of NEC, BPD and IVH in extremely preterm neonates.

Al Tawil et al [99]	2013	Saudi Arabia	Retrospective case control	150	32	NEC development prediction according to CBC results	PLT counts	The presence of thrombocytopenia was associated with a poor prognosis and increased risk of mortality (OR=33.6, 95%: 3.43–328.9)
Gaudin et al [80]	2013	France	Retrospective	60	60	Stricture development in association with multiple baseline clinical and laboratory characteristics	PLT	The occurrence of post-NEC stricture was associated significantly with the presence of thrombopenia (<100000 platelets/mm3).
Miner et al [64]	2013	USA	Retrospective multicenter study	220	220	Association of NEC severity with laboratory parameters	Hct, WBC, neutrophil, lymphocyte, PLT, I/T, MPV	Preterm infants with earlier gestational age, lower birth weight, previous RBC transfusions, absence of early colostrum feedings, acidosis, abnormal CBC, elevated CRP, and sepsis are at higher risk of developing severe NEC requiring surgery.
Atici et al [63]	2014	Turkey	prospective cohort study	31	31	NEC prognosis based on clinical and laboratory parameters	hemoglobin, hematocrit, white blood cell, neutrophil, lymphocyte and platelet counts	Thrombocytopenia was the only CBC parameter related to mortality. Optimal cut-off level was 110.000/μL (with 93.3% sensitivity and 87.5% specificity AUC=0.838, 95% CI: 0.667-1.008; p=0.001).
Remon et al [38]	2014	USA	Retrospective case control	326	69	To assess whether an acute decrease in peripheral blood monocyte counts could differentiate early NEC from other causes of feeding intolerance	WBC, neutrophil, and monocyte counts	Monocyte counts were significantly lower in neonates with both Bell stage II and III disease on the day of onset of NEC and in the 1st follow-up CBC. A fall in peripheral blood AMC was a useful diagnostic marker of NEC in VLBW infants. An acute drop in AMC (from the last available test) at the time of feeding intolerance onset correctly discriminated NEC cases from other causes of feeding intolerance with 76% accuracy.
Sho et al [33]	2014	USA	Retrospective case control	157	157	Ability of laboratory and clinical parameters to predict NEC totalis	Complete blood count (WBC, RBC, hemoglobin, hematocrit, MCV, MCH, MCHC, RDW, MPV, platelets), differential and absolute blood counts (neutrophils, bands, lymphs, monocytes, basophils, eosinophils, atypical lymphs, metamyelocytes, myelocytes and nucleated RBCs)	The presence of thrombocytopenia was the strongest independent risk factor and was associated with over a 84-fold higher risk of NEC-totalis (OR=84.3,95 %: [2.67, 2670].
Okur et al [86]	2015	Turkey	Retrospective case control	330	30	Comparison of platelet mass index between NEC and controls	PLT, PMI,MPV first and second measure	Lower platelet mass index values between 3 and 7 days (second measure) were recorded in VLBW neonates with NEC (p = 0.011) when compared to the control group.
Wahidi et al [60]	2015	USA	Retrospective study	90	40	To assess the connection between sustained blood eosinophilia and medical or surgical complications in NEC	Eosinophils	Early persistent eosinophilia (defined as ≥5% of the total leukocyte count for ≥5 consecutive days following the onset of NEC) was a risk factor for all studied adverse outcomes such as intestinal strictures and/or liver fibrosis.
Christensen et al[57]	2015	USA	Retro case control	3650	NR	NEC development among others morbidities	WBC, neutrophil counts I/T, PLT	Neutropenia was independently associated with a higher risk of acquiring NEC (odds ratio [OR] 4.01, 95% CI 2.08–7.35, P , .001). Thrombocytopenia alone (without neutropenia) was not associated with higher risk of acquiring NEC (OR 1.16, 95% CI 0.72–1.79, P = .60).
Banerjee et al [61]	2015	UK	Retrospective	890	195	To investigate if the hemoglobin level at birth is linked to short-term outcomes in preterm infants born at or before 32 weeks of gestation.	Hb	Hb at birth was not a statistically significant factor for NEC when adjusted for BW and GA. Increased Hb level at birth by delaying umbilical cord clamping has been demonstrated to reduce the risk of NEC
Patel et al [46]	2016	USA	Prospective, multicenter observational cohort study	598	44	To investigate the association between RBC transfusion, severe anemia, and the occurrence of NEC.	Hemoglobin levels	VLBW neonates with severe anemia (hemoglobin level lower than 8 g/dL) at a given week, had a higher estimated rate of NEC compared to those without severe anemia (adjusted cause-specific HR, 5.99 [95% CI, 2.00-18.0]; P = .001). RBC transfusion did not correlate with the overall incidence of NEC.
Gordon et al [103]	2016	USA	Retrospective cohort study	5166	5166	NEC mortality predicted by CBC parameters on the day of diagnosis	Hct-Hb-PLT-WBC-segmented neutrophils-and bands, lymphocytes-Eosinophils (percentages and absolute counts)	In neonates who died the total WBC, absolute neutrophil count and segmented neutrophils and bands (Segs/bands) were higher; but the hemoglobin/hematocrit, absolute monocyte count, absolute eosinophil count and platelet count were lower, compared to those who lived. In a multivariate analysis: decreased PLT counts, higher absolute MON-LYMPH count, Segs/bands >0.2 were identified as the most important hematologic factors associated with death. Low platelet counts (≤150 000) and severe anemia at NEC onset were associated with a higher risk of mortality across all gestational ages.
Yu et al [50]	2016	China	Retrospective	57	57	To determine risk factors and clinical outcomes for bowel perforation in preterm neonates	PLT, WBC,	The perforated NEC group had lower PLT counts and more abnormal WBC counts (<5 or >20× 109/L) in comparison to the non perforated NEC group.
Zhang et al[88]	2017	China.	Retrospective multicenter study	188	186	Stricture development in association with multiple baseline clinical and laboratory data	WBC, PLT, Hb,plateletcrit at disease onset	Neonates with stricture exhibited significantly elevated levels of C-reactive protein (CRP), white blood cells (WBC), and plateletcrit , with these increased levels persisting until the stricture resolved.
Yang et al [31]	2018	China.	Cross sectional study	161	103	The diagnostic potential of the neutrophil/lymphocyte (NRL) ratio for the early detection of neonatal NEC was assessed.	WBC count, neutrophil/lymphocyte ratio	Strong correlations were observed between the NLR and other indicators in NEC stages II and III, with the N/L ratio demonstrating higher sensitivity, specificity, and Youden index compared to WBC count, CRP, and PA, suggesting that it is a better continuous marker for the early diagnosis and severity differentiation of NEC
Luo et al[58]	2019	China	Retrospective control study	58	29	Difference in PLT counts among NEC and controls	PLT	Platelet counts (129.9 ± 25.4 × 109/L) in NEC patients were significantly lower compared to the non-NEC group (249.7 ± 39.5 × 109/L) ($P < 0.05$). The combination of resistin-like molecule β (RELMβ) and thrombocytopenia resulted in the detection of neonatal NEC with AUC: 0.841 ($P < 0.05$) with a sensitivity of 82.9% and a specificity of 93.2%, respectively.
Lin et al [51]	2019	China	Retrospective	352	352	To identify clinical and laboratory factors risk factors related to neonatal fulminant NEC and to develop a scoring system to identify patients at risk for NEC-totalis at the time of presentation.	Hct, PLT, WBC, neutrophil counts	To identify clinical and laboratory factors that differentiate NEC-totalis from other types of NEC and to create a scoring system to identify patients at risk for NEC-totalis at the time of presentation.
Desiraju et al [105]	2020	USA	Retrospective study	143	76	The ability to distinguish eventual Stages 2 and 3 from the percent delta AMC calculated at illness onset.	Absolute monocyte count	No significant AMC change from baseline is seen in infants with rule-out NEC or bacteremia without NEC. Infants with Stages 2 and 3 NEC show significant changes in AMC at the time of illness onset, relative to baseline ($p < 0.001$). Infants with Stage 3 NEC demonstrate a change in monocyte count that is significantly greater than the change seen in Stage 2 NEC, $p < 0.001$. There is a significant difference between the AMC change in rule-out NEC versus true NEC (Stages 2 or 3), $p < 0.001$.
Cai et al [36]	2020	China	Retrospective study	80	11	NEC development prediction according to CBC results.	PLT,WBC, Hb	The decrease in hemoglobin concentration, and rates of red blood cell transfusion and ventilator application were significantly higher in the NEC group than in the non-NEC group (all $P < 0.05$), while no significant differences in the WBC count, platelet count, and hemoglobin concentration, and blood culture were noted among the two groups.

Alhamad et al [73]	2020	USA	Retrispective cohort	185	37	To investigate whether complete blood counts (CBCs) and differentials at the onset of feeding intolerance (FI) enhance early identification of NEC or sepsis.	absolute neutrophil count (ANC), AMC, the platelet count, and the I/T ratio	CBC parameters failed to distinguish between NEC and sepsis. The combination of the presence of a change in platelet count of ≥10% and an I/T ratio of ≥ 0.1 at the onset of FI provided a fair-to-good diagnostic accuracy for NEC or sepsis (AUC 0.74, with a sensitivity 0.50 and a specificity of 0.97)
Go et al [107]	2020	Japan	Retrospective	305	10	NEC development (among others, IVH-BPD, mortality)	PLT, PMI,MPV, PCT	No CBC parameters were served as prognostic factors of NEC.
Haefeli et al [98]	2020	Switzerland	Retro case control	78	26	To identify risk factors for NEC in neonates with a significant patent ductus arteriosus (PDA)	Hb, WBC, PLT before NEC onset	NEC patients had lower Apgar scores (1'), higher incidence of congenital malformations, higher minimum platelet counts, and elevated CRP values prior to NEC onset, with higher mortality rates (29% vs. 2%, P < 0.001),
Kordazs et al [97]	2021	Switzerland	Retrospective multicenter study	157	157	Difference in CBC parameteres between survivors-non survivors, severe - non severe NEC	CBC parameters assessed	Low Hgb levels correlated with severe NEC and mortality. A proportion of immature neutrophils above 34% at disease onset was found to be associated with NEC stage III (OR 2.9, 1.2–7.4, P 0.025), while a WBC count higher than 22G/l during the course of disease was correlated with severe NEC (OR 4, 1.8–9.3, P < 0.001).
Su et al [54]	2021	China	Prospective study	86	86	Difference in CBC parameters between cases of mild and severe NEC	WBC, PLT, neutrophil, lymphocytes	The levels of WBC in neonates with severe NEC were significantly higher than in those with mild NEC (p <0.001). NEC severity was found to be positively correlated with WBC counts (r=0. 946, p <0.001), negatively correlated with PLT counts (r=-0. 602, p <0.001), and had a weak correlation with neutrophils and lymphocytes (r=0. 186, p=0. 087; r=0. 072, p=0. 509).
Ibrohim et al [69]	2021	Indonesia	Retrospective study	214	214	To determine the prognostic factors associated with clinical deterioration in preterm neonates diagnosed with NEC.	leukocyte count, and platelet count	In this cohort, leukocytosis and thrombocytopenia were not reliable predictors of clinical deterioration of NEC.
Zheng et al [30]	2021	China	Retrospective case control	200	93	The role of platelet-to-lymphocyte ratio (PLR) in early diagnosis of NEC in preterm neonates	PLT/lymphocyte ratio (PLR)	PLR value >100 within 7 days before NEC diagnosis was found to be an independent risk factor for NEC [OR: 18.82 (95% CI: 2.93–120.98), p = 0.002].
Raba et al [106]	2021	ireland	Retrospective study	59	59	To examine the value of laboratory parameters in the differentiation between medical and surgical NEC	white blood cell (WBC) and platelet count at 24-48-72 h after the diagnosis)	Platelet count and leukocyte levels decreased by 72 h post-diagnosis in the surgical group, but these differences were not significant.
Siahaan et al [84]	2021	Indonesia	Retrospective study	52	52	To assess survival of neonates with NEC and associate it with the prognostic factors	PLT	Platelet count was not significantly associated with the survival of neonates with NEC.
Pantalone et al [67]	2021	USA	Retrospective case control	246	177	To evaluate the GA-specific immune abnormalities during the course of NEC through a comprehensive analysis of the CBC differential.	white blood cell (WBC) count, absolute monocyte count (AMC), absolute lymphocyte count (ALC), absolute eosinophil count (AEC), absolute neutrophil count (ANC), and absolute band count at NEC diagnosis and percent change from baseline	The CBC differential may be used to identify the neonates at high risk of requiring surgical intervention.
Song J et al [65]	2021	China	Retrospective study	447	296	A novel metaheuristic algorithm was proposed to predict NEC diagnosis and prognosis.	white blood cell count, lymphocyte percentage, and mean platelet volume, Hb.	A feature selection and classification algorithm using pre-disease data for diagnostic classification and NEC risk prediction was developed. Neutrophil percentage, breast milk, probiotics, MCH, and anemia-RBC transfusion were identified as key predictors for classic and surgical NEC, playing a significant role in early diagnosis and risk assessment.
Feng et al [40]	2022	China	Retrospective study	114	114	To evaluate the potential of PLT count to predict NEC surgery and mortality.	PLT, WBC, Hb	Surgical NEC was significantly associated with decreased WBC counts (median: 8.93 versus 10.19 [× 109/L], P = 0.041), HB levels (mean: 133.20 versus 146.03 [g/L], P = 0.035), and PLT counts (median: 163.0 versus 328.0 [× 109/L], P < 0.001). PLT counts were identified as an independent predictor for the need for surgery in NEC patients (OR = 0.995, 95% CI: 0.990 ~ 0.999, P = 0.029; AUC: 0.763).
Qin et al [44]	2022	China	retrospective cohort study	157	157	Risk factors of severe surgical NEC and mortality.	absolute neutrophil count (ANC), before and at the onset of NEC, difference in absolute neutrophil count (ΔANC) at NEC onset, and platelet counts	A decrease in neutrophil count was the most sensitive predictive factor for severe surgical NEC and death, especially when combined with PLT counts. Even when adjusted for multiple confounders for each 109/L ΔANC reduction at NEC onset, the odds for severe NEC increased by almost 25% (OR 1.308, 95% CI 1.113–1.539; P = 0.001).
Feng et al [55]	2022	China	Retrospective study	131	131	To evaluate the association of SII with surgical risk in neonates with NEC.	WBC, neutrophil count, NLR, PLR,SII	A prediction model based on the combination of Low-SII and Low-PLR resulted in an AUC of 0.838 (95% CI: 0.764–0.897, P < 0.001) indicating a good predictive performance for identifying the patients who received surgical intervention.
Elmoneim et al [92]	2022	S. Arabia	Retrospective	188	NR	To assess the risk factors for the development of NEC and other co morbidities.	PLT counts , PLT counts drop >30% within 7 days	The odds of having NEC were significantly higher (P <0.01) in preterm neonates who had ≥ 30% decrease in platelet counts either with or without thrombocytopenia than those of thrombocytopenic preterm neonates with no decline in platelets decline.
Tajalli et al [77]	2022	Iran	Case -control	160	80	To evaluate and compare blood monocyte counts in preterm neonates with NEC and those without the diagnosis.	WBC, Neutrophils, Lymphocytes, Monocytes	The receiver operating characteristic of AMC values demonstrated a diagnostic power of 0.693 (95% CI: 0.612–0.773) for NEC and 0.738 (95% CI: 0.627–0.850) for stage II and III NEC.
Han et al [39]	2022	China	Retrospective	271	271	To examine the surgical outcomes in neonates with perforated versus non-perforated NEC and determine the criteria for surgical intervention.	PLT, WBC	CBC parameters were similar among 2 groups (perforated and non-perforated group). Infants with surgical NEC in the non-perforated group were more prone to bowel necrosis, and their mortality rate was higher than that in the perforated group.
Liu et al[21]	2022	China	Prospective study	60	60	To investigate the predictive value of certain laboratory metrics in forecasting the deterioration of NEC.	neutrophiI, I/T-PLT	Neonates in the NEC Stage III group, presented with significantly lower neutrophil counts compared to neonates with NEC stage II with an AUC of neutrophil 0.666.
Li et al[93]	2022	China	Retrospective stydy	207	207	To identify predictors for bowel resection in neonates diagnosed with NEC.	CBC parameters assessed	An increased incidence of neutropenia [p=0.004]) at disease diagnosis in neonates with NEC was associated with bowel loss, suggesting a severe case of NEC. Neutrophil, counts on day 1 after NEC onset along with other parameters (birth weight < 2520g, hypotension, pneumoperitoneum, acidosis, and intestinal wall thickness >1.08mm) were identified as most key variables and were included in a predictive model for severe NEC.

Diez et al [56]	2022	Germany	Retrospective	28	28	To investigate the role of DMBT1 expression in relation to cardiac status and its association to fulminant course of NEC.	NLR and MLR.	Both ratios (NLR and MLR) were significantly higher in infants with persisting PDA and CHD in comparison to infants with normal cardiac anatomy (p = 0.0319 and 0.0493, respectively).
Garg et al [52]	2022	USA	Retrospective	209	209	To identify the risk factors and outcomes associated with sepsis in neonates with NEC	WBC, neutrophil, PLT	Neonates with NEC-associated sepsis had a lower absolute lymphocyte count on the day of NEC onset and a lower median platelet count at 24 hours after NEC onset (p=0.031) compared to those without NEC-associated sepsis. PLTs were significantly lower in the sepsis group, both at onset and at 24 h after diagnosis.
Cai et al [72]	2022	China	Retrospective cohort	75	75	To evaluate the role of the MPV and procalcitonin PCT in predicting the severity of necrotizing enterocolitis NEC in preterm infants	CBC parameters assessed	MPV and PCT were significantly higher in neonates with severe NEC in comparison to those with mild-moderate NEC; however the WBC count was lower in the former. No significant differences in PLT, RDW, Hb and CRP were observed between the two groups (P > 0.05). The results from the logistic regression suggested that the MPV (OR = 6.194, P = 0.000 <0.05) and PCT (OR = 1.093, P = 0.006 <0.05) were independent predictive factors of the severity of NEC and MPV combined with PCT was superior in predicting the severity of NEC (AUC: 0.895, P<0.000).
Song J et al [101]	2022	China,	case control	467	467	To investigate the relationship between severe anemia, red blood cell transfusions, and the development of NEC in neonates.	Hemoglobin	In very low birth weight (VLBW) neonates, after adjusting for other variables, severe anemia within 72 hours (OR = 2.404, P = 0.016), RBC transfusion within 24 hours (OR = 4.905, P = 0.016), within 48 hours (OR = 5.587, P = 0.008), and within 72 hours (OR = 2.858, P = 0.011) were associated with an increased risk of developing NEC.
Garg et al [72]	2022	USA		336	336	To assess whether hematological profiles and transfusion patterns following the onset of NEC can help identify infants at risk of developing severe, fatal NEC.	CBC, hematocrit, hemoglobin, PLT	Neonates with fulminant NEC often exhibited thrombocytopenia, lymphopenia, neutropenia, and leukopenia. Additionally, those who received red blood cell transfusions after NEC onset or platelet transfusions before its onset were more likely to develop the fulminant form of the disease.
Mu and Wang [45]	2022	China	Retrospectrive case control	199	93	Diagnosing NEC using the NLR ratio	WBC, NLR	An NLR value between ≥ 1.60 and < 3.20 within 1 week prior to NEC diagnosis could significantly reduce the risk of preterm NEC (OR, 0.10; 95% CI, 0.00–0.40; P < 0.001)
Kasirer et al [85]	2023	Israel	Retrospective case control	141	63	Difference in platelet indices between the NEC and control group	Platelet count, MPV, platelet distribution width and RPR and delta platelets from birth to the time of NEC diagnosis or day of life 14 in the control group.	All platelet indices measured—except of MPV—were significantly associated with NEC diagnosis. Although MPV was not associated with the diagnosis of NEC (p = 0.800), it was significantly associated with NEC-related mortality (p < 0.001). Only the total platelet count and RPR were significantly associated with both NEC diagnosis (p < 0.0001) and mortality (p = 0.04 and 0.01, respectively). On multivariable analysis only the change in platelet count from birth to the time of diagnosis was significantly associated with NEC.
Chen et al [90]	2023	China	Retrospectve	216	216	To develop a prediction model of the rapid progression (Rp) of NEC in preterm neonates.	white blood cell count, hemoglobin , neutrophil count	White blood cell count <5*10^9/L, hemoglobin <100 g/L, neutrophil count <2*10^9/L, pH <7.3, and abnormal coagulation were positively correlated with RP-NEC in the invariable regression analysis, while in multivariable regression NEUT<2.000 was the only CBC parameter difference between the two groups (most common in RpNEC).
Yu et al [87]	2023	China	Retrospective	267	267	To explore the high-risk factors for surgical NEC.	erythrocytes, hemoglobin, white blood cells, platelets	Lower leukocytes (P = 0.001), lymphocytes (P < 0.001), erythrocytes (P = 0.004), and platelets counts (P = 0.039) were noted in neonates with surgical NEC. The multivariate logistic regression analysis identified lymphocytes counts as a potentially protective factor for surgical NEC (OR = 0.749; 95% CI: 0.588-0.954; P = 0.019).
Li et al [82]	2023	China	Retrospective	206	206	To develop and assess a predictive nomogram for FNEC.	Hb, WBC, neutrophil, lymphocyte, eosinophils and monocyte counts at 3 intervals: 24 h before, at onset, 24 h after NEC	Neutrophil counts on the day of NEC onset as well as neutrophil, lymphocyte, and monocyte counts on day 1 after NEC onset along with other parameters (assisted ventilation after NEC onset, shock at NEC onset) were identified as the most relevant variables and were included in a predictive model for FNEC, which exhibited good discrimination capacity (AUC: 0.884; 95% CI 0.825–0.943).
Jiang et al [42]	2023	China	Retrospective cohort	155	155	To asses if the sepsis, anemia, and PLT activation index are vital NEC predictors in LBW neonates.	Hb levels, PLT indicators (such as PLT count, PDW , MPV, plateletcrit , and PLCR)	In LBW neonates without sepsis, anemia [P = 0.001, odds ratio (OR) = 4.367, 95% confidence interval (CI): 1.853–10.291], high PLCR values (P < 0.001, OR = 2.222, 95% CI: 1.633–3.023), and high PCT values (P =0.024, OR = 1.368, 95% CI: 1.042–1.795) increased the risk of NEC; AUC of PLCR, sensitivity, specificity, and cutoff value were 0.739, 0.770, 0.610, and 33.55, respectively.
Zouari et al [89]	2023	Tunisia	Retrospective study	102	102	To evaluate the predictive factors for mortality in patients with NEC.	CBC, PLT	Gestational age < 32 weeks, Apgar score<8 at 5 minutes, very low birth weight, severe thrombocytopenia, Bell's stage 3 and sepsis during hospitalization were identified as predictive factors for mortality in neonates with NEC.
Dantes et al [53]	2024	USA	Retrospective cohort study	338	69	To examine the clinical characteristics associated with SIP and NEC diagnosis and develop a scoring algorithm for accurate preoperative diagnosis.	CBC parameters	The differences in CBC parameters were not statistically significant in aiding the differentiation between SIP and surgical NEC. A risk score was developed using statistically significant parameters (pneumatosis, abdominal wall erythema, higher ALD and history of feeds)
Moroze et al [49]	2024	USA	Retrospective cohort study	130	130	To assess the potential of AMC trends over 72 hours in suspected NEC.	AMC	A decrease in AMC may serve as a useful biomarker for confirming NEC diagnosis, particularly in differentiating stage 2/3 from lower-risk cases.
Guo et al [41]	2024	China	Retrospective study	191	191	To assess the predictive value of CBC parameters, CRP, and PCT in determining the severity of NEC, and to develop a model for distinguishing surgically treated NEC from non-surgically treated NEC.	PLR and the combination of WBC, ANC, ALC, NLR	Elevated PLR is linked to severe inflammation in patients with surgical or fatal NEC. The predictive model combining ANC, PLR, CRP, and PCT can distinguish surgical/fatal NEC from medical NEC.
Huang et al [94]	2024	China	Retrospective study	160	160	To investigate the clinical characteristics of NEC complicated by intestinal perforation, identify associated risk factors, develop effective early predictors, and construct a visual scoring system for independent risk variables to provide a scientific basis for reducing morbidity and mortality in neonates with NEC.	PLT count	Thrombocytopenia and hypoalbuminemia may serve as independent risk factors for predicting intestinal perforation in neonates with NEC.
Assenga and Tooke [79]	2024	South Africa	retrospective observational cohort	1582	104	To assess the proportion, patterns, and risk factors associated with mortality in VLBW neonates diagnosed with NEC in a middle-income setting.	complete blood count	Anaemia necessitating blood transfusion (p = 0.003) and thrombocytopenia requiring platelet transfusion (p = 0.033) were identified as significant factors linked to increased mortality in NEC cases.

Zhang et al [29]	2024	China	Retrospective study	372	372	To explore the potential role of MPV and PDW in predicting surgical neonatal NEC, and to establish the relationship between MPV/PDW levels and the severity or prognosis of NEC.	blood cell count analysis, MPV and PDW	MPV and PDW could serve as useful indicators for assessing the need for surgical intervention and predicting the prognosis in patients with NEC.
Chong et al [68]	2024	China	Retrospective study	229	229	To identify age-specific hematological biomarkers that could predict surgical NEC.	WBC, hemoglobin; Hct, PLT, ANC, absolute lymphocyte count; absolute monocyte count; absolute eosinophil count; ABC: absolute basophil count	Patients with NEC show distinct hematological characteristics based on GA, and independent predictors of surgical NEC vary across different GAs.
Li et al [93]	2024	China	Retrospective study	334	334	To develop and establish a predictive model for RP-NEC.	CBC parameters	Plasma sodium levels <135 mmol/L, C-reactive protein ≥10 mg/L, platelet count <100 × 10^9/L, lymphocyte count <1.5 × 10^9/L, blood pH <7.2, and the presence of ascites at the onset of NEC were identified as independent risk factors for RP-NEC and were incorporated in the predictive model, which demonstrated an AUC value of 0.983 (95% CI 0.97–0.99).
Meng et al [48]	2024	China	Retrospective study	122	122	To evaluate the predictive value of rintSO_2 combined with PCT and MPV in determining the severity of NEC in preterm infants.	WBC, PLT, PCT, MPV, RDW, Hb, CRP	The combination of rintSO_2, PCT, and MPV may serve as early biomarkers for assessing NEC severity, aiding in early diagnosis and timely intervention to improve prognosis.

Abbreviations: Absolute Lymphocyte Count, ALC; Absolute Eosinophil Count, AEC; Absolute Monocyte Count, AMC; Absolute Neutrophil Count, ANC; Area Under the Curve, AUC; Base Excess, BEs; Complete Blood Count, CBC; Confidence Interval, CI; C-Reactive Protein, CRP; Congenital Heart Defects, CHD; Feeding Intolerance, FI; Fulminant Necrotizing Enterocolitis, FNEC; Gestational Age, GA; Hazard Ratio, HR; Hematocrit, Hct; Hemoglobin, Hb; Immature/Total Neutrophil Ratio, I/T; Intraventricular Hemorrhage, IVH; Intestinal Tissue Oxygen Saturation, rintSO_2; Low Birth Weight, LBW; Malignant Brain Tumor 1, DMBT1; Mean Corpuscular Hemoglobin, MCH; Mean Corpuscular Hemoglobin Concentration, MCHC; Mean Corpuscular Volume, MCV; Mean Platelet Volume, MPV; Monocyte-to-Lymphocyte Ratio, MLR; National Cancer Institute, NCI; Necrotizing Enterocolitis, NEC; Neutrophil/Lymphocyte Ratio, NLR; Nucleated Red Blood Cell, NRBC; Odds Ratio, OR; Patent Ductus Arteriosus, PDA; Platelet Distribution Width, PDW; Platelet Mass Index, PMI; Platelet-Neutrophil, PN; Platelet-to-Lymphocyte Ratio, PLR; Platelets, PLT; Procalcitonin, PCT; Proportion of Large Platelets, PLCR; Rapidly Progressive Necrotizing Enterocolitis, RpNEC; Red Blood Cell, RBC; Red Cell Distribution Width, RDW; Red Cell Distribution Width to Platelet Ratio, RPR; Resistin-Like Molecule Beta, RELMβ; Spontaneous Intestinal Perforation, SIP; Systemic Immune-Inflammation Index, SII; Very Low Birth Weight, VLBW; White Blood Cell, WBC.
